# Supplementary material for: Universal exploration dynamics of random walks
Source: Nat Commun. 2023 Feb 4;14:618. doi: 10.1038/s41467-023-36233-5 (PMC9899275; doi:10.1038/s41467-023-36233-5)
Supplement: Supplementary file 1 — Supplementary Information [file 41467_2023_36233_MOESM1_ESM.pdf]

SUPPLEMENTARY INFORMATION
Universal exploration dynamics of random walks

Léo Régnier, Maxim Dolgushev, S. Redner, and Olivier Bénichou

CONTENTS

S1. Scaling form of the distribution $F_n(\tau)$ for a nearest-neighbor random walk in $1d$ 2

S2. Distribution $Q_n(r)$ of the radius of the largest spherical region free of traps after n sites have been visited 2
- A. Typical radius ρ_n for marginal RWs 2
  - 1. Theoretical argument 2
  - 2. Numerical check of the hypotheses. 4
- B. Typical radius ρ_n for recurrent RWs 6
- C. Typical radius ρ_n for transient RWs 6
- D. Scaling form of $Q_n(r)$ 7

S3. Analytical expressions for $F_n(\tau)$ in Table I of the main text 8
- A. Short-time behavior of $F_n(\tau)$: algebraic decay 8
  - 1. Equation (1) of the main text 8
  - 2. Solution to Eq. (1) of the main text 9
  - 3. The exponent of the algebraic decay: The first moment approach 10
  - 4. Prefactor of the algebraic decay 10
- B. Long-time behavior of $F_n(\tau)$: exponential and stretched exponential regimes 11
- C. Scaling form $F_n(\tau)$ for recurrent RWs, Eq. (4) of the main text 12
- D. Moments of τ_n 12
  - 1. Analytical derivation 12
  - 2. Numerical check of the moments 13

S4. Numerical simulations 14
- A. Recurrent random walks 14
- B. Exact enumeration technique 14
- C. Monte Carlo simulations of transient RWs 15
  - 1. Importance sampling: general idea 15
  - 2. Choice of the energy 16
  - 3. The Monte Carlo moves: example 16
  - 4. Choice of the bias: Wang-Landau 18
  - 5. Results of the Monte Carlo simulations 18
  - 6. Stretched exponential without the Monte Carlo method 19

S5. Extensions 19
- A. General observables 19
  - 1. Definition of bulk and boundary observables 19
  - 2. Dynamics of bulk observables 19
  - 3. Dynamics of boundary observables 19
- B. Covariance of the number of distinct sites visited for recurrent $1d$ Lévy flights 21
  - 1. Derivation of the multiple time correlations 21
  - 2. Criterion on τ_n correlations 22
- C. Non-Markovian RWs 25
  - 1. CTRW 25
  - 2. $1d$ Lévy flights with crossing 26
  - 3. Lévy walks 28
  - 4. Algebraic decay for non-Markovian recurrent random walks 28

Supplementary References 29

S1. SCALING FORM OF THE DISTRIBUTION $F_n(\tau)$ FOR A NEAREST-NEIGHBOR RANDOM WALK IN $1d$

In $1d$, τ_n is the exit time from an interval of length $L = n$ starting at a distance $a = 1$ from the edge of the interval. The Laplace transform in the continuous limit is well known [S1], and given by

$$\widehat{F}_L(s) = \frac{\sinh\left(\sqrt{\frac{s}{D}}a\right) + \sinh\left(\sqrt{\frac{s}{D}}(L-a)\right)}{\sinh\left(\sqrt{\frac{s}{D}}L\right)} , \tag{1}$$

where D is the diffusion coefficient. Then in the limit $L \to \infty$, $s \to 0$ with sL^2 fixed, we obtain

$$\widehat{F}_L(s) \sim 1 - \sqrt{\frac{s}{D}} a \tanh\left(\sqrt{\frac{s}{D}}\frac{L}{2}\right) . \tag{2}$$

By using residue calculus, and replacing a by 1, L by n, and setting $D = 1/2$, we obtain

$$F_n(t) \sim \frac{2\pi^2}{n^3} \sum_{k=0}^{\infty} (2k+1)^2 \; e^{-\pi^2(2k+1)^2 t/2n^2} , \tag{3}$$

which we may re-express in the following scaling form as written in the introduction of the main text,

$$F_n(t) \sim n^{-3} \psi\left(\frac{t}{n^2}\right) . \tag{4}$$

S2. DISTRIBUTION $Q_n(r)$ OF THE RADIUS OF THE LARGEST SPHERICAL REGION FREE OF TRAPS AFTER n SITES HAVE BEEN VISITED

We determine the typical value ρ_n of the radius of the largest spherical region completely covered by a RW, when n distinct sites have been visited. We make use of the result for the mean of the number of distinct sites visited $N(t)$ at time t by a RW (neglecting prefactors of order 1) [S2, S3]:

$$\langle N(t) \rangle = n \sim \begin{cases} t^{\mu}, & \mu \equiv d_{\mathrm{f}}/d_{\mathrm{w}} < 1 \qquad \text{(recurrent RWs)} \\ t/\ln t, & \mu = 1 \qquad \text{(marginal RWs)} \\ t, & \mu > 1 \qquad \text{(transient RWs)} . \end{cases} \tag{5}$$

A. Typical radius ρ_n for marginal RWs

1. *Theoretical argument*

We start with the marginal case, and follow the approach of [S4]. There, it was shown that the size of the largest ball entirely covered by a $2d$ random walker after t steps typically behaves as $\rho_t = t^{1/4}$ (up to logarithmic prefactors). As we will show, this result can be extended to any marginal random walk and is given by $\rho_t = t^{1/2d_{\mathrm{f}}}$ (up to logarithmic prefactors). This result is equivalent to the statement that the radius r of the largest ball entirely covered by a marginal random walker before exiting this ball is typically $\sqrt{r}$, using that $r \sim t^{1/d_{\mathrm{w}}}$ by the definition of d_{w}.

The strategy is to split the ball of radius r into disjoint balls of radius r^{γ} with $0 < \gamma < 1$, and determine the largest exponent γ such that at least one ball of radius r^{γ}/e^2 [S5] has been fully visited before exiting the ball of radius r. Consider a ball of radius r (Supplementary Figure 1). It contains $r^{d_{\mathrm{f}}-\gamma d_{\mathrm{f}}}$ disjoint balls of radius r^{γ}. First, we ask how many times the random walk will make an incursion inside a ball of radius r^{γ} before exiting the ball of radius r. To answer this question, we use that the splitting probability of hitting a circle of radius a before hitting the circle of radius b, when starting at distance ρ from the center, is [S1]

$$\pi(\rho) = \frac{\ln(\rho/b)}{\ln(a/b)} . \tag{6}$$

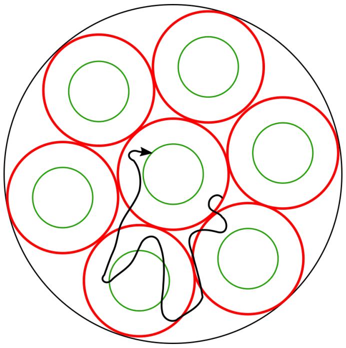

Supplementary Figure 1. Representation of a γ-incursion in $2d$ (black curve with an arrow): a trajectory starting from a red sphere of radius r^γ reaching the green sphere of radius r^γ/e within the same sphere before the black sphere of radius r.

We say that the walker, which starts from the surface of the ball of radius $\rho = r^\gamma$, makes a γ-incursion inside the ball of radius r^γ whenever it hits the ball of radius $a = r^\gamma/e$ before a ball of radius $b = r$ (Supplementary Figure 1). The probability of such an incursion is

$$\frac{\ln(r^\gamma/r)}{\ln(r^\gamma/(e\,r))} \sim 1 - \frac{1}{(1-\gamma)\ln r} \ .$$

Thus the probability of making k such incursions is

$$\mathbb{P}(X = k) = \left(1 - \frac{1}{(1-\gamma)\ln r}\right)^k \sim \exp\left(-\frac{k}{(1-\gamma)\ln r}\right) \tag{7}$$

for one ball of radius r^γ. That is, the distribution of the number of incursions is exponentially distributed.

Now we assume that the number of γ-incursions for all of the $M = r^{d_\mathrm{f}-\gamma d_\mathrm{f}}$ balls are iid random variables X_m $(m = 1, \ldots, M)$, that are exponentially distributed, with average $\langle X \rangle = (1-\gamma)\ln r$. Thus the average maximum number of γ-incursions $\mathcal{M}_M = \max X_m$ is given by

$$\begin{aligned}
\langle \mathcal{M}_M \rangle &= \int_0^\infty \mathbb{P}\left(\mathcal{M}_M \geq k\right) \mathrm{d}k \\
&= \int_0^\infty \left(1 - \mathbb{P}\left(X < k\right)^M\right) \mathrm{d}k \\
&= \int_0^\infty \left(1 - \left[1 - \exp\left(-k\langle X \rangle\right)\right]^M\right) \mathrm{d}k \\
&= \int_0^\infty \sum_{i=0}^{M-1} \left[1 - \exp\left(-k/\langle X \rangle\right)\right]^i \exp\left(-k/\langle X \rangle\right) \mathrm{d}k \\
&= \langle X \rangle \int_0^1 \sum_{i=0}^{M-1} (1-x)^i \,\mathrm{d}x \sim \langle X \rangle \ln\left(M\right) \ .
\end{aligned} \tag{8}$$

Replacing M and $\langle X \rangle$ by their values above, we find that there are at most $d_\mathrm{f}(1-\gamma)^2(\ln r)^2$ incursions inside a ball of size r^γ.

We now ask how many incursions inside a ball of radius r^γ are necessary to visit all the sites within the ball of radius r^γ/e^2. We start by computing the probability to reach the origin when performing a γ-incursion, considering that the incursion stops when the RW hits the sphere of radius r^γ (at which an other γ-incursion may occur). Using Eq. (6) for the splitting probability for an outer radius $b = r^\gamma$, inner radius $a = 1$, and the radius of the starting position $\rho = r^\gamma/e$ (start of the γ-incursion), we obtain the probability to reach the origin during this incursion to be $1/(\gamma \ln r)$. Thus the probability of not reaching the origin during any of k γ-incursions is

$$\left(1 - \frac{1}{\gamma \ln r}\right)^k \sim \exp\left(-\frac{k}{\gamma \ln r}\right) \ .$$

Because there are $(r^\gamma/e^2)^{d_\mathrm{f}}$ sites inside a ball of radius r^γ/e^2, we estimate the probability of having at least one non-visited site after k incursions to be

$$\begin{aligned}
&1-\mathbb{P}(\text{all sites are visited in the ball of radius } r^\gamma/e^2)\\
&\approx 1-\mathbb{P}(0 \text{ is visited})^{(r^\gamma/e^2)^{d_\mathrm{f}}}\\
&=1-\left(1-\exp\left(-\frac{k}{\gamma\ln r}\right)\right)^{(r^\gamma/e^2)^{d_\mathrm{f}}}\\
&\approx (r^\gamma/e^2)^{d_\mathrm{f}}\times\exp\left(-\frac{k}{\gamma\ln r}\right)=\frac{1}{e^{2d_\mathrm{f}}}\exp\left(\gamma\, d_\mathrm{f}\,\ln r-\frac{k}{\gamma\ln r}\right),
\end{aligned} \tag{9}$$

by assuming that all $(r^\gamma/e^2)^{d_\mathrm{f}}$ sites are equivalent to the origin, and exploration of all these specific sites are independent events. This probability goes to zero for $k > k_c(r) = \gamma^2 d_\mathrm{f}\,(\ln r)^2$. Thus $\gamma^2 d_\mathrm{f}\,(\ln r)^2$ incursions are necessary to visit all the sites within the ball of radius r^γ.

Finally, since there are at most $(1-\gamma)^2\, d_\mathrm{f}(\ln r)^2$ incursions inside a ball of radius r^γ and that $\gamma^2\, d_\mathrm{f}\,(\ln r)^2$ incursions are necessary to visit the entire ball of radius r^γ/e^2, equating these two numbers gives

$$\gamma = 1/2. \tag{10}$$

Thus

$$\rho_n = \sqrt{r} \sim t^{1/2d_\mathrm{w}} \sim n^{1/2d_\mathrm{f}}\ , \tag{11}$$

up to logarithmic corrections. This is the scaling behavior of ρ_n for marginal RWs written in the main text.

2. *Numerical check of the hypotheses.*

We check numerically the different assumptions which lead to the scaling obtained in the previous part, by relying marginal $1d$ Lévy flights (for which $\rho_n \sim n^{1/2}$). We start by checking that the $\left(r^\gamma/e^2\right)^{d_\mathrm{f}}$ sites are equivalent to the origin, in the sense that they have the same probability to be visited by the kth incursion as the origin. We compute numerically the probability that a site x has been visited at the kth $\gamma-$incursion inside a domain of radius r/e (taking $\gamma=1$, as here this exponent plays no role), starting at $\pm r/e$ and stopping the incursion when the ball hits the radius r. We denote this probability by $p_{x,r}(k)$. Then, we check that $p_{x,r}(k)$ is independent of the site x. In Supplementary Figure 2, we take different values of x and r and verify that, indeed, at radius r large enough, the visitation statistics at the kth incursion is independent of the site x.

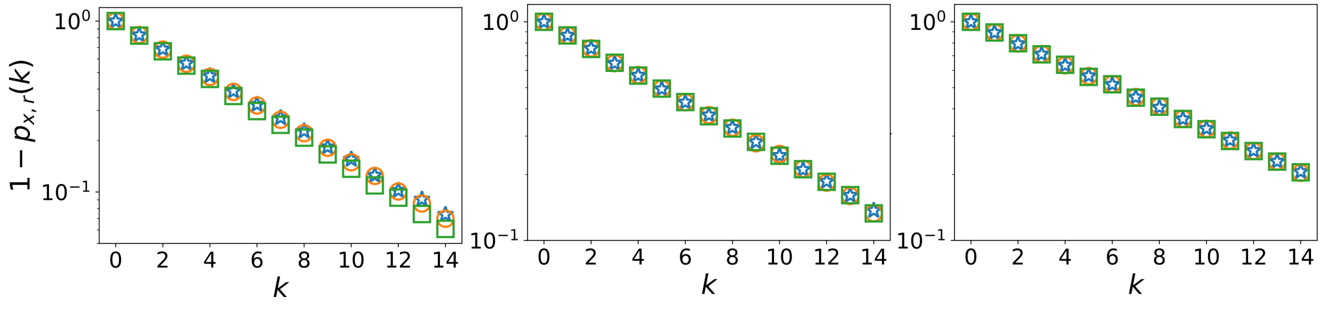

Supplementary Figure 2. Distribution $1-p_x,r(k)$ of the probability not to have visited site x among the k $\gamma-$incursions ($\gamma=1$) inside a ball of radius $r=100/e$, $r=1000/e$ and $r=10000/e$ (from left to right), and for $x=0$, $x=10$, $x=20$ (blue stars, orange circles, and green squares, respectively).

We then confirm the hypothesis on the independence of sites by measuring in numerical simulations the correlation in the visitation events. More precisely, we look at the probability of having visited site 0 and site x before or at the $k^{\mathbf{th}}$ $\gamma-$incursion inside a ball of radius r/e ($\gamma=1$, the model is the same as for the first part of this answer and the exponent still plays no role). We denote this probability by $p_{0\cap x,r}(k)$. In particular, we looked at the cases $k=1$ and $k=10$. Then, the independence is verified if $p_{0\cap x,r}(k) \approx p_{0,r}(k)p_{x,r}(k)$. Because for large values of k

$p_{0\cap x,r}(k) \approx p_{0,r}(k) \approx p_{x,r}(k) \approx 1$, we say that the visitation events are effectively independent when the relative difference

$$C_r^k(x) = \frac{|p_{0\cap x,r}(k) - p_{0,r}(k)p_{x,r}(k)|}{1 - p_{0\cap x,r}(k)} \tag{12}$$

is smaller than a threshold value (0.1 for example). In Supplementary Figure 3, we show that for increasing values of r, for both $k = 1$ and $k = 10$, the fraction of sites x whose visitation is effectively independent of the visitation of site 0 increases. This is a direct consequence of the decrease of $C_r^k(x)$ with both x/r and r.

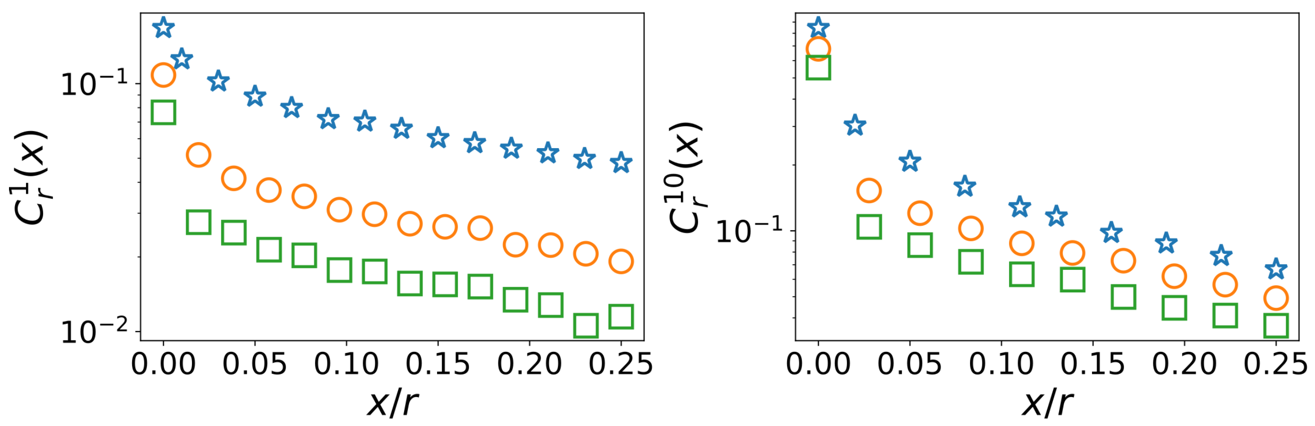

Supplementary Figure 3. Relative difference $C_r^k(x)$ of the simultaneous visitation probability of site 0 and site x for $k = 1$ (left) and $k = 10$ (right), for γ−incursions ($\gamma = 1$) inside a ball of radius $r = 10^2/e$, $r = 10^4/e$, and $r = 10^6/e$ (blue stars, orange circles, and green squares, respectively).

Finally, the equivalence and the independence hypotheses lead to the scaling $\rho_n \propto n^{1/2}$ for the marginal case. Additionally, we check the scaling of $k_c(r)$ with r in Supplementary Figure 4, $k_c(r)$ being the mean number of incursions starting at $\pm r/e$ in a ball of radius r necessary to have visited all the sites inside the ball of radius r/e^2. The $(\ln r)^2$ asymptotics is confirmed, even though the convergence occurs only at large values of r.

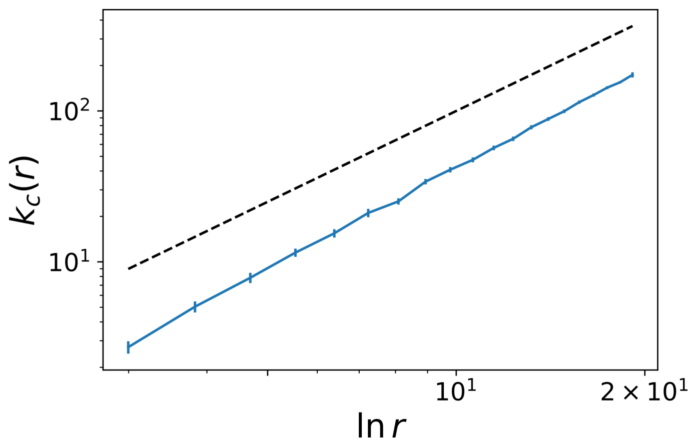

Supplementary Figure 4. $k_c(r)$ compared to $(\ln r)^2$ in black dashed line for a $1d$ Lévy flight of parameter $\alpha = 1$. Errors bars are the 95% confidence intervals.

B. Typical radius ρ_n for recurrent RWs

The splitting probability in the previous subsection is, in case of recurrent RWs [S6, S7],

$$1-\pi(\rho)\propto\left(\frac{\rho}{b}\right)^{-\Delta}\ , \tag{13}$$

where, for notational simplicity, $\Delta \equiv d_{\mathrm{f}} - d_{\mathrm{w}} < 0$ and a is taken to 0 as for recurrent random walks the size of a target is not relevant to determine the probability of it being reached. Hence, the probability of making k incursions in the ball of radius r^γ is $\exp\left(-kr^{\Delta(1-\gamma)}\right)$. Thus the maximum number of incursions is $\ln(r^{d_{\mathrm{f}}(1-\gamma)})\ r^{-\Delta(1-\gamma)} = d_{\mathrm{f}}(1-\gamma)\ r^{-\Delta(1-\gamma)}\ \ln r$, by following similar steps to those used to derive Eq. (8).

Using Eq. (13) for the splitting probability for an outer radius $b = r^\gamma$ and the radius of the starting position $\rho = r^\gamma/e$, we obtain $\pi(\rho) \sim \text{const.} \equiv \pi_0$. The probability π_0^k of not seeing the origin during any of the k incursions is then independent of r. Because there are $(r^\gamma/e^2)^{d_{\mathrm{f}}}$ sites inside the ball of radius r^γ/e^2, we estimate the probability of having at least one non-visited site when k incursions have occurred as $(r^\gamma/e^2)^{d_{\mathrm{f}}} \times \pi_0^k$, by asserting that all sites are equivalent to the origin, and that the exploration of sites are independent events. This probability goes to 0 for $k > k_c(r) = \gamma d_{\mathrm{f}}\ \ln r/\ln(1/\pi_0)$. Note that for large r, the number of incursions required before visiting every site ($\propto \gamma \ln r$) is much smaller than the expected maximum number of incursions in a ball ($\propto (1-\gamma)\ r^{-\Delta(1-\gamma)}\ \ln r$). This means that $\gamma = 1$. These considerations lead to

$$\rho_n = r \sim t^{1/d_{\mathrm{w}}} = n^{1/d_{\mathrm{f}}}\ . \tag{14}$$

C. Typical radius ρ_n for transient RWs

For transient walks, the splitting probability is [S6, S7]:

$$\pi(\rho) = 1 - \frac{1-\left(\dfrac{a}{\rho}\right)^{\Delta}}{1-\left(\dfrac{a}{b}\right)^{\Delta}}\ . \tag{15}$$

When $\rho \to \infty$, $\pi(\rho) \sim \text{const.} \equiv \pi_1$. Thus the probability of making k incursions into a ball of radius r^γ is π_1^k, and thus the maximum number of incursions is $\ln(r^{d_{\mathrm{f}}(1-\gamma)})/\ln(1/\pi_1)$.

Using Eq. (15) for the splitting probability for an outer radius $b = r^\gamma$, inner radius $a = 1$, and the radius of the starting position $\rho = r^\gamma/e$, we obtain the probability of not reaching the origin during any of these k incursions:

$$(1-\pi(r^\gamma/e))^k = \left(1-\frac{e^\Delta - 1}{r^{\gamma\Delta}-1}\right)^k \sim \exp\left(-k\frac{e^\Delta-1}{r^{\gamma\Delta}}\right)\ .$$

Because we have to visit $(r^\gamma/e^2)^{d_{\mathrm{f}}}$ sites, the random walk must take

$$k > k_c(r) = \gamma d_{\mathrm{f}}\ \frac{r^{\gamma\Delta}}{e^\Delta - 1}\ \ln r$$

steps to ensure that all sites have been visited. We observe that the number of incursions to visit every site inside the ball of radius r^γ/e^2 is much larger than the expected maximum number of incursions into a ball ($\propto (1-\gamma)\ln r$ vs. $\propto \gamma r^{\gamma\Delta}\ln r$, respectively). This means that $\gamma = 0$, and the typical size of a region entirely visited by a transient walk when exiting the domain of size r is smaller than any power law in r or in n,

$$\rho_n = O(r^0) = O(n^0)\ . \tag{16}$$

Indeed, as we will show in the following, the radius of the largest ball for which every site is visited grows as $\rho_n \sim (\ln t)^{1/\Delta} \sim (\ln n)^{1/\Delta}$ (because the number of visited sites grows linearly with the number of steps, we do not distinguish between ρ_n defined with n the number of distinct sites visited or ρ_t with t the number of steps). First, we cut the trajectory of the RW of length n into non-overlapping balls of radius r. There are approximately $n/r^{d_{\mathrm{w}}}$ such balls, because the fractal dimension of the visited domain is d_{w}. For each of these balls, indexed by i, we are interested in the time t_i that the RW spends inside the ball before exiting. Because the walk is transient, we suppose

that once the RW exits the ball, it never returns. For each of the balls, the exit time is exponentially distributed (at long times) and its average is proportional to $r^{d_{\rm w}}$ [S2]. Now we make the approximation that the exit times t_i are independent, and we obtain that the maximal value for t_i is given by $t_{\max}(n) \propto r^{d_{\rm w}} \ln\left(n/r^{d_{\rm w}}\right)$, as a consequence of Eq. (8). Using that the RW is transient, the number of distinct sites visited by the RW up to time $t_{\max}(n)$ is proportional to $t_{\max}(n)$. Thus at least one ball of radius r has been fully visited as soon as

$$t_{\max}(n) > r^{d_{\rm f}} \; . \tag{17}$$

Thus the optimal radius ρ_n, which corresponds to r such that $t_{\max}(n) = r^{d_{\rm f}}$, is

$$\rho_n = (\ln n)^{1/\Delta} \; . \tag{18}$$

Ref. [S8] has proven this result for RWs with nearest-neighbor jumps ($d_{\rm w} = 2$) on a hypercubic lattices in dimension $d \geq 3$. Equation (18) is also compatible with the previous prediction that ρ_n grows more slowly than any power of n.

D. Scaling form of $Q_n(r)$

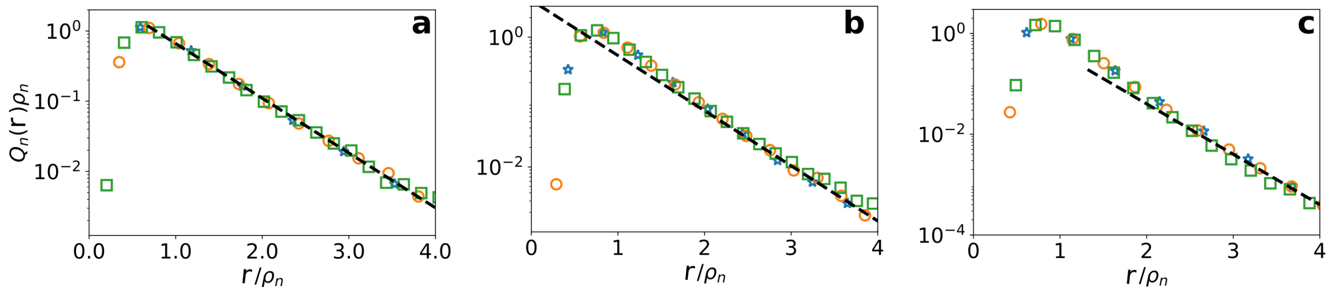

Supplementary Figure 5. Distribution of the radius of the largest trap-free domain for Lévy flights in 1d, with the radius rescaled by the average value ρ_n for: **a** $\alpha = 1.2$ ($n = 1600,\ 3200,\ 6400$ in blue, orange, and green, respectively), **b** $\alpha = 1$ ($n = 400,\ 800,\ 1600$ in blue, orange, and green, respectively), and **c** $\alpha = 0.8$ ($n = 200,\ 800,\ 3200$ in blue, orange, and green, respectively).

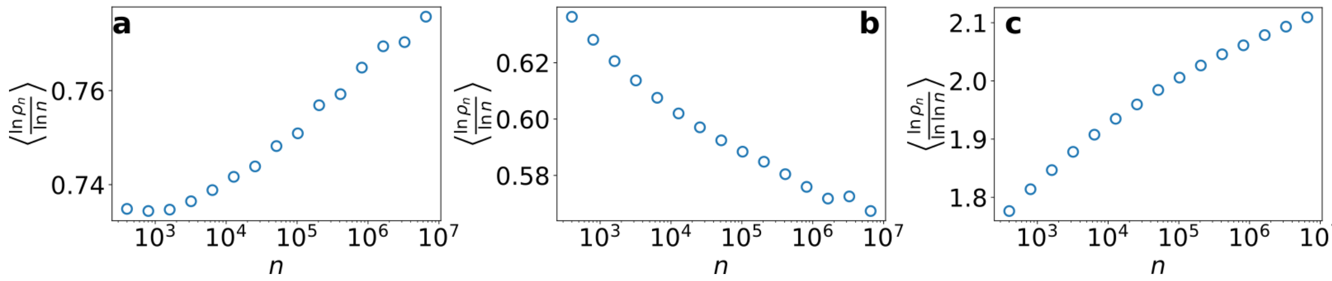

Supplementary Figure 6. Evolution of ρ_n with n, defined as the radius of the largest fully visited ball within a visited domain of n sites, for Lévy flights with parameters: **a** $\alpha = 1.2$, **b** $\alpha = 1$, and **c** $\alpha = 0.8$.

From the typical length scale ρ_n determined above, we argue that the probability density $Q_n(r)$ of the radius of the largest spherical trap-free region free is

$$Q_n(r) = \frac{1}{\rho_n} f\left(r/\rho_n\right) . \tag{19}$$

The characteristic length ρ_n can be interpreted as a correlation length, so that two sites separated by a distance larger than ρ_n have effectively independent visitation probabilities. Similar to the celebrated idea of independent "blobs"

defined in [S9, S10], we consider that the RW represents a polymer that can be separated into independent blobs. Within each blob of radius ρ_n, all the sites are either all visited or not visited at all, and the visitation probabilities between distinct blobs are independent. For large values of r/ρ_n, this leads to an exponential distribution:

$$Q_n(r) \approx \frac{1}{\rho_n}\, e^{-a(r/\rho_n)^{d_\mathrm{f}}} \;, \tag{20}$$

since $(r/\rho_n)^{d_\mathrm{f}}$ is the number of independent domains free of traps/fully visited blobs. Here a is a constant that is independent of r and n.

We confirm the scaling form as well as the asymptotic exponential decay in Supplementary Figure 5. Supplementary Figure 6 displays the extraordinarily slow approach of ρ_n to the asymptotic formulas derived above. These results are illustrated for Lévy flights for the three main cases. For recurrent ($\alpha = 1.2$), marginal ($\alpha = 1$), and transient ($\alpha = 0.8$) flights, we use Eqs. (14), (11), (18) to predict that $\ln\rho_n/\ln n \to 1$, $\ln\rho_n/\ln n \to 1/2$, and $\ln\rho_n/\ln\ln n \to 5$, respectively.

S3. ANALYTICAL EXPRESSIONS FOR $F_n(\tau)$ IN TABLE I OF THE MAIN TEXT

A. Short-time behavior of $F_n(\tau)$: algebraic decay

1. *Equation (1) of the main text*

Similar to the approach given in [S11], we start by deriving a renewal equation for the exit-time distribution, conditioned on the visited domain consists of n sites, whose list of positions is denoted by the vector $\mathbf{r}_n$. We set the position of the RW at $\tau = 0$ to be x. For the list of previously visited sites $\mathbf{r}_n$ and starting position x, we denote $F_n(\tau', x'|\mathbf{r}_n, x)$ as the first-passage probability to site x' at time τ' before any reaching other of the non-visited sites. The quantity $P_\mathrm{trap}(\tau|\mathbf{r}_n,\ x)$ is defined as the probability to be at a non-visited site at time τ, conditioned on the visited domain being $\mathbf{r}_n$ and the starting position of the RW is x at time $\tau = 0$. Partitioning this last event over the first-passage at site x' at time τ', we obtain

$$P_\mathrm{trap}(\tau|\mathbf{r}_n,\ x) = \delta(\tau) + \sum_{\tau'=1}^{\tau}\sum_{x'\notin\mathbf{r}_n} F_n(\tau', x'|\mathbf{r}_n, x) P_\mathrm{trap}(\tau-\tau'|\mathbf{r}_n, x') \;. \tag{21}$$

Here we make the approximation that since x is close to the surface of $\mathbf{r}_n$, we take the starting position x to be at a trap. To perform averages over the initial site x, we introduce $P(\mathbf{r}_n, x)$ as the joint probability for the RW to visit the set of sites $\mathbf{r}_n$ and is at position x at time $\tau = 0$, so that the quantity $P_\mathrm{trap}(\tau)$ we are interested in can be written as

$$P_\mathrm{trap}(\tau) \equiv \langle P_\mathrm{trap}(\tau|\mathbf{r}_n,\ x)\rangle = \sum_{\mathbf{r}_n, x} P_\mathrm{trap}(\tau|\mathbf{r}_n,\ x) P(\mathbf{r}_n, x) \;. \tag{22}$$

Using this definition, we have

$$\begin{aligned} P_\mathrm{trap}(\tau) &= \delta(\tau) + \sum_{\tau'=1}^{\tau}\left\langle \sum_{x'\notin\mathbf{r}_n} F_n(\tau', x'|\mathbf{r}_n, x) P_\mathrm{trap}(\tau-\tau'|\mathbf{r}_n, x')\right\rangle \\ &\approx \delta(\tau) + \sum_{\tau'=1}^{\tau} \langle F_n(\tau'|\mathbf{r}_n, x) P_\mathrm{trap}(\tau-\tau'|\mathbf{r}_n)\rangle \\ &\approx \delta(\tau) + \sum_{\tau'=1}^{\tau} F_n(\tau') P_\mathrm{trap}(\tau-\tau') \;. \end{aligned} \tag{23}$$

In the mean-field type formula above, we neglected the dependence of $P_\mathrm{trap}(\tau-\tau'|\mathbf{r}_n, x') \approx P_\mathrm{trap}(\tau-\tau'|\mathbf{r}_n)$ on the starting site x' (keeping in mind only that $x' \notin \mathbf{r}_n$) and the spatial correlations in the visited domain. Numerical simulations (see early-time algebraic regime of Fig. 3**a**, **b**, and **c** of the main text and of Supplementary Figure 9**a**) justify the applicability of (23). For early times τ and for visited domains of large size n, $F_n \approx F_\infty$, and we finally get Eq. (1) of the main text.

2. Solution to Eq. (1) of the main text

To solve this equation, we first need to determine the time dependence of $P_{\text{trap}}(t)$. From Eq. (2) of the main text, $P_{\text{trap}}(t) \propto t^{(d_{\text{T}}-d_{\text{f}})/d_{\text{w}}}$, where d_{T} is the fractal dimension of the interface between visited and non-visited sites. The problem is then reduced to specifying d_{T}. For this, we determine the time evolution of the number of sites $P(t)$ on the interface between visited and non-visited sites for recurrent and marginal RWs at time t. By interpreting the set of $\langle \tau_n \rangle$ as the average discovery rate of new sites, we relate the perimeter $P(t)$ of the visited domain (the number of traps) and the size of the visited domain $V(t)$:

$$\frac{\mathrm{d}V(t)}{\mathrm{d}t} \sim \frac{1}{\langle \tau_{V(t)} \rangle} \; . \tag{24}$$

Using Kac's lemma [S12], the average return time to the boundary of the visited domain is

$$\langle \tau_{V(t)} \rangle \propto \frac{V(t)}{P(t)} \; . \tag{25}$$

Combining Eqs. (24) and (25) leads to

$$P(t) \sim \frac{\mathrm{d}}{\mathrm{d}t} \left(V(t)^2 \right) \; . \tag{26}$$

Using the results of Eq. (5), with $n = V(t)$, we get

$$P(t) \sim \begin{cases} t^{2d_{\text{f}}/d_{\text{w}}-1} & d_{\text{f}} < d_{\text{w}} \\ t/\ln^2 t & d_{\text{f}} = d_{\text{w}} \; , \end{cases} \tag{27}$$

which gives the fractal dimension of the surface (neglecting logarithmic prefactors):

$$d_{\text{T}} = 2d_{\text{f}} - d_{\text{w}}, \quad \text{for } d_{\text{f}} \leq d_{\text{w}} \; . \tag{28}$$

We verify the time dependence of the perimeter of the visited domain in Supplementary Figure 7 for three types of recurrent random walks. We note that the result for the $2d$ random walk is already known from [S13] and for $1d$ Lévy flights as well [S14]. We note that in Kac's lemma the perimeter of the domain $D(t)$ of volume $V(t)$ would be defined as

$$P(t) = \sum_{i \notin D(t)} p(i \to j) \; . \tag{29}$$

For nearest neighbour random walks it is straightforwardly proportional to the perimeter defined in the usual manner. For Lévy flights, we verify that indeed it is similar by using this definition for the perimeter in Supplementary Figure **7a**, and see that the scaling is the same as in [S14]. Using this result, we obtain the scaling behaviour $P_{\text{trap}}(t) \propto t^{\mu-1}$.

We now relate P_{trap} and F_∞, which is most conveniently done in the Laplace domain. Defining the Laplace transform of a function $f(t)$ by $\mathcal{L}\{f(\tau)\} \equiv \widehat{f}(s) = \int_0^\infty f(t)e^{-st}dt$, Eq. (1) of the main text leads to

$$\widehat{P}_{\text{trap}}(s) = 1 + \widehat{F}_\infty(s)\widehat{P}_{\text{trap}}(s),$$

which yields

$$\widehat{F}_\infty(s) = 1 - \frac{1}{\widehat{P}_{\text{trap}}(s)} \; . \tag{30}$$

Using $P_{\text{trap}}(t) \propto t^{\mu-1}$, we have $\widehat{P}_{\text{trap}}(s) \propto 1/s^\mu$.

Finally, differentiating Eq. (30) with respect to s (to get a diverging quantity as $s \to 0$), we obtain

$$\mathcal{L}\{\tau F_\infty(\tau)\} = -\frac{\mathrm{d}\widehat{F}_\infty(s)}{\mathrm{d}s} = \frac{\mathrm{d}}{\mathrm{d}s}\left(\frac{1}{\widehat{P}_{\text{trap}}(s)} \right) \propto s^{\mu-1} \; .$$

From the Tauberian theorem [S15] that links the small-s asymptotics in the Laplace domain to the long-time asymptotics, we finally obtain the algebraic decay of $F_\infty(\tau)$ that is written in the main text,

$$F_\infty(\tau) \propto \tau^{-1-\mu} \; . \tag{31}$$

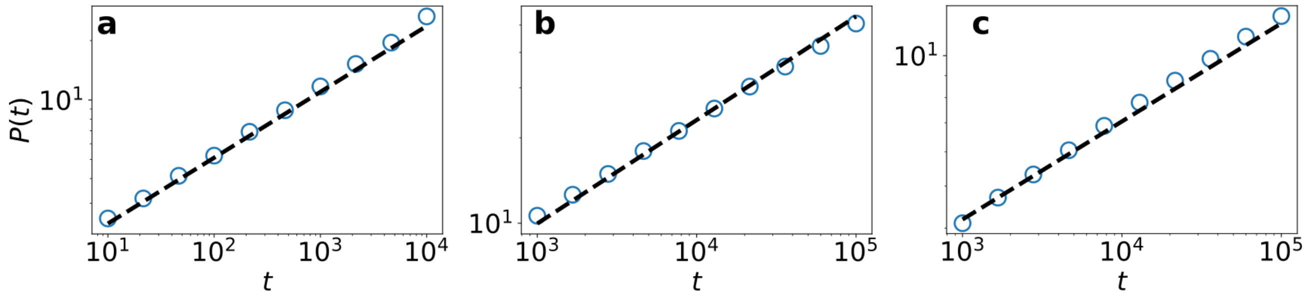

Supplementary Figure 7. Average perimeter $P(t)$ of the visited domain for **a** 1d Lévy flights of parameter $\alpha = 1.5$, **b** nearest-neighbor random walks on a Sierpinski gasket **c** nearest-neighbor random walks on a T-tree. The black dashed line represents the prediction (27).

3. *The exponent of the algebraic decay: The first moment approach*

We present here an alternative approach that allows one to recover the exponent δ that characterizes the algebraic decay of $F_n \propto \tau^{-1-\delta}$ determined in Eq. (31). This approach consists of identifying two expressions for $\langle \tau_n \rangle$. On the one hand, the very definition of $\langle \tau_n \rangle$ gives

$$\langle \tau_n \rangle \propto \int_1^{t_n} \frac{\tau}{\tau^{1+\delta}} \, \mathrm{d}\tau \propto t_n^{1-\delta} = n^{(1-\delta)/\mu} \; . \tag{32}$$

On the other hand, knowing that $\langle \tau_n \rangle$ is the time scale needed to discover a new site,

$$\frac{\mathrm{d}\, \langle N(t) \rangle}{\mathrm{d}t} \sim t^{\mu-1} \sim \frac{1}{\left\langle \tau_{n=\langle N(t) \rangle} \right\rangle}. \tag{33}$$

Using finally the temporal scaling of the number of distinct sites visited (see Eq. (5)), $n \sim t^\mu$, we again find $\delta = \mu$.

4. *Prefactor of the algebraic decay*

This subsection is devoted to the analysis of the prefactor $A(\mu)$ in the algebraic decay that appears in Fig. 3 of the main text.

Exact value for marginal RWs. In the marginal case ($\mu = 1$), we can obtain the prefactor exactly. We exploit the fact that there are two independent ways to express $\langle \tau_n \rangle$. The first method relies on the self-averaging property of $N(t)$ in which $\mathrm{Var}(N(t))/\langle N(t) \rangle^2 \to 0$ as $t \to \infty$ (see [S2, S14]). Thus the number of distinct sites visited any marginal RW is close to its average value, which scales asymptotically as $\beta t / \ln t$, with β depending on the specific RW model. We now use the link between the distributions of $N(t)$ and $\sum_{k \le n} \tau_k$:

$$\mathbb{P}\left(N(t) \ge n\right) = \mathbb{P}\Big(\sum_{k \le n} \tau_k \le t \Big) \approx \Theta\left(\beta t / \ln t - n\right) \approx \Theta\left(t - n \ln n / \beta\right) \; , \tag{34}$$

with Θ the Heaviside function, which is 0 for negative argument and 1 otherwise. This last equation shows that $\sum_{k \le n} \tau_k$ also has negligible fluctuations and is peaked at the value $(n \ln n)/\beta = \sum_{k \le n} \langle \tau_k \rangle$. Thus

$$\langle \tau_n \rangle \sim \frac{\ln n}{\beta} \; . \tag{35}$$

The second method relies on the algebraic decay $F_n(k) = A(\mu = 1) k^{-2}$ for $k < t_n$, to compute the asymptotic behavior of the first moment:

$$\langle \tau_n \rangle = \sum_{k=1}^{\infty} k F_n(k) \sim \sum_{k=1}^{t_n} \frac{A(1)}{k} + O(1) \sim A(1) \ln t_n \sim \tfrac{1}{2} A(1) \ln n \; , \tag{36}$$

where we used that $t_n \propto \sqrt{n}$ at large times. We note that the times $k > t_n$ do not contribute to the average, because of the rapid stretched exponential decay of $F_n(k)$. Identifying the expressions for $\langle \tau_n \rangle$ in the previous two equations, we obtain the exact value

$$A(1) = \frac{2}{\beta} \tag{37}$$

in the marginal case.

We now use the known facts that $\beta = \pi$ for the nearest-neighbor random walk on the square lattice [S3] and $\beta = 3$ for Lévy flights in $1d$ and parameter $\alpha = 1$ [S3, S14]. Thus we obtain $F_n(\tau)$, including the correct amplitude, for marginal RWs for $1 \ll \tau_n \ll \sqrt{n}$:

$$F_n(\tau) \sim \begin{cases} \dfrac{2}{\pi\tau^2} & \text{for nearest-neighbor RWs on square lattice} \\ \dfrac{2}{3\tau^2} & \text{for Lévy flights in } 1d,\ \alpha = 1\ . \end{cases} \tag{38}$$

Recurrent RWs. We obtain an approximate value of the prefactor $A(\mu)$ in the algebraic regime from the normalization condition

$$1 = \sum_{k=1}^{\infty} F_n(k) = \sum_{k=1}^{t_n} \frac{A(\mu)}{k^{1+\mu}} + \sum_{k>t_n} \frac{\text{const}}{n^{1/\mu+1}}\, e^{-\text{const}\ k/n^{1/\mu}} \sim A(\mu)\zeta(1+\mu) + O\left(\frac{1}{n}\right)\ , \tag{39}$$

from which we infer $A(\mu) = 1/\zeta(1+\mu)$.

B. Long-time behavior of $F_n(\tau)$: exponential and stretched exponential regimes

Here we obtain the stretched exponential and exponential decays at long times. We start from Eq. (3) of the main text, written for $\tau > t_n$,

$$S_n(\tau) \geq \frac{q_n}{\rho_n} \int_0^{n^{1/d_{\rm f}}} e^{-b\tau/r^{d_{\rm w}} - a(r/\rho_n)^{d_{\rm f}}} \mathrm{d}r\ , \tag{40}$$

where $q_n \equiv \int_{t_n}^{\infty} F_\infty(\tau')\mathrm{d}\tau'$ and a and b are constants. To determine the large-τ behavior of the integral on the rhs of Eq. (40), we apply the Laplace method. It is convenient to first make the variable change variable $r = \rho\,\tau^{\mathcal{D}}$, where again, for notational simplicity, $\mathcal{D} \equiv 1/(d_{\rm w} + d_{\rm f})$, to give

$$S_n(\tau) \geq \frac{q_n \tau^{\mathcal{D}}}{\rho_n} \int_0^{n^{1/d_{\rm f}}/\tau^{\mathcal{D}}} \exp\left[-\tau^{d_{\rm f}\mathcal{D}}\, U(\rho)\right] \mathrm{d}\rho\ , \tag{41}$$

with $U(\rho) \equiv b/\rho^{d_{\rm w}} + a(\rho/\rho_n)^{d_{\rm f}}$. To proceed with the Laplace method, the location of the minimum of $U(\rho)$, $\rho^* \sim \rho_n^{d_{\rm f}\mathcal{D}}$ must be smaller than $n^{1/d_{\rm f}}/\tau^{\mathcal{D}}$. Equivalently, $r^*(\tau) = \rho^*\,\tau^{\mathcal{D}} \sim \rho_n(\tau/t_n)^{\mathcal{D}} < n^{1/d_{\rm f}}$. This condition means that $\tau < n^{1+1/\mu}/\rho_n^{d_{\rm f}} \equiv T_n$. Thus, for times $t_n < \tau < T_n$, the Laplace method gives

$$S_n(\tau) \geq \frac{q_n \tau^{\mathcal{D}}}{\rho_n} \sqrt{\frac{2\pi}{U''(\rho^*)\tau^{\mu/(1+\mu)}}}\ \exp\left[-\tau^{\mu/(1+\mu)}\, U(\rho^*)\right]\ . \tag{42}$$

Because

$$\rho^* \sim \rho_n^{d_{\rm f}\mathcal{D}} = \rho_n^{\mu/(1+\mu)} \qquad \text{and} \qquad U(\rho^*) \sim \rho_n^{-\mu d_{\rm w}/(1+\mu)} = t_n^{-\mu/(1+\mu)}\ ,$$

we obtain for $t_n < \tau < T_n$:

$$S_n(\tau) \geq \text{const}\ \times q_n \tau^{\mathcal{D}-\mu/2(1+\mu)} \rho_n^{(d_{\rm f}-2)/2(1+\mu)}\ \exp\left[-\text{const}\,(\tau/t_n)^{\mu/(1+\mu)}\right]\ . \tag{43}$$

For $\tau > T_n$ and ρ in the range $[0, n^{1/d_{\rm f}}/\tau^{1/(d_{\rm w}+d_{\rm f})})]$, $U(\rho)$ has its minimum at the upper bound $n^{1/d_{\rm f}}/\tau^{\mathcal{D}}$. Thus we are left with

$$S_n(\tau) \geq q_n\ \exp\left[-b\tau/n^{1/\mu} - an/\rho_n^{d_{\rm f}}\right]\ . \tag{44}$$

Now we study the explicit form of this lower bound for each class of RWs (recurrent, marginal and transient), following the results of section S2 about the scalings of ρ_n with n. In addition, as in the classic trapping problem, we expect that this lower bound for the survival probability will have the same time dependence as the survival probability itself.

- recurrent RWs. Here $\mu < 1$ and $\rho_n \sim n^{1/d_\mathrm{f}}$. Because $t_n = n^{1/\mu}$ and $T_n = n^{1+1/\mu}/\rho_n^{d_\mathrm{f}} = n^{1/\mu}$, there is no intermediate regime and we have

$$S_n(\tau) \approx q_n \, \exp\left[-b\tau/n^{1/\mu} - a\right] , \tag{45}$$

with $q_n = \int_0^{n^{1/\mu}} \mathrm{d}\tau/\tau^{1+\mu} \propto 1/n$. This leads, for $\tau \gg t_n$, to

$$F_n(\tau) \propto \frac{1}{n^{1+1/\mu}} \exp\left[-b\tau/n^{1/\mu}\right] . \tag{46}$$

- marginal RWs. Here $\mu = 1$ and $\rho_n \sim n^{1/2d_\mathrm{f}}$, so that $t_n = \sqrt{n}$, $T_n = n^{3/2}$ and $q_n \propto 1/\sqrt{n}$. Thus we expect for the intermediate regime (up to algebraic prefactors in τ and n)

$$S_n(\tau) \approx e^{-\mathrm{const}\times\sqrt{\tau/\sqrt{n}}} , \tag{47}$$

which results in the stretched exponential behavior of Table I. For the exponential regime, we get

$$F_n(\tau) \propto \frac{1}{n^{3/2}} \, e^{-b\tau/n - a\sqrt{n}} . \tag{48}$$

- transient RWs. Here $\mu > 1$ and $\rho_n \sim 1$ (neglecting logarithmic corrections). This behavior for ρ_n leads to $t_n = 1$ (i.e., there is no algebraic regime), $T_n = n^{1+1/\mu}$, and $q_n \propto 1$. We expect for the intermediate regime (up to algebraic prefactors in τ and n)

$$S_n(\tau) \approx e^{-\mathrm{const}\times\tau^{\mu/(1+\mu)}} , \tag{49}$$

which results in the stretched exponential behavior of Table I. For the exponential regime, we get

$$F_n(\tau) \propto \frac{1}{n^{1/\mu}} \, \exp\left[-b\tau/n^{1/\mu} - an\right] . \tag{50}$$

C. Scaling form $F_n(\tau)$ for recurrent RWs, Eq. (4) of the main text

Here we show that the distribution $F_n(\tau)$ for recurrent RWs admits the scaling form Eq. (5) of the main text. As shown above, $F_n(\tau)$ displays two regimes for recurrent RWs: algebraic (Eq. (31)) and exponential (Eq. (46)). Noting that the algebraic regime can be rewritten as $F_n(\tau) \propto \tau^{-1-\mu} = \frac{1}{n^{1+1/\mu}}\left(\tau/n^{1/\mu}\right)^{-1-\mu}$, both regimes can be synthesized into the scaling form:

$$F_n(\tau) = \frac{1}{n^{1+1/\mu}} \, \psi\left(\frac{\tau}{n^{1/\mu}}\right) . \tag{51}$$

The scaling function $\psi(x)$ decays algebraically for small x and exponentially for large x.

D. Moments of τ_n

1. *Analytical derivation*

Moments of τ_n follow from Table I of the main text. For recurrent walks ($\mu < 1$), the scaling form (51) yields

$$\langle \tau_n^k \rangle = \sum_{\tau=1}^{\infty} \frac{\tau^k}{n^{1/\mu+1}} \psi\left(\frac{\tau}{n^{1/\mu}}\right) \sim n^{k/\mu-1} \int_0^\infty u^k \psi(u) \mathrm{d}u . \tag{52}$$

For marginal walks ($\mu = 1$), we use the result that the algebraic regime holds up to $\tau \propto t_n = \sqrt{n}$ and gives the scaling behavior of the moments as

$$\langle \tau_n^k \rangle \propto \sum_{\tau=1}^{\sqrt{n}} \frac{\tau^k}{\tau^2} = \sum_{\tau=1}^{\sqrt{n}} \tau^{k-2} \, , \tag{53}$$

which results in $\langle \tau_n \rangle \propto \ln n$ and $\langle \tau_n^k \rangle \propto n^{(k-1)/2}$ for $k > 1$.

For transient walks ($\mu > 1$), both the number of visited sites and the number of sites on the interface between visited and non-visited site grow linearly with time [S14]. Then Eq. (2) in the main text gives $P_{\text{trap}}(\tau)$ is a constant t, and we we denote this constant as c. Thus the early-time first-passage probability to a trap has the exponential form

$$F_n(\tau) = (1-c)^{\tau-1} c \, . \tag{54}$$

If this exponential regime breaks down at a time $t_n = (\ln n)^{1/\Delta}$ which diverges as $n \to \infty$, then we have

$$\langle \tau_n^k \rangle \sim \sum_{\tau=1}^{t_n} \tau^k F_n(\tau) \sim \sum_{\tau=1}^{\infty} \tau^k (1-c)^{\tau-1} c = \text{const} \, . \tag{55}$$

2. *Numerical check of the moments*

Here we present the results of the numerical simulations and verify the predicted moments obtained in the previous paragraph.

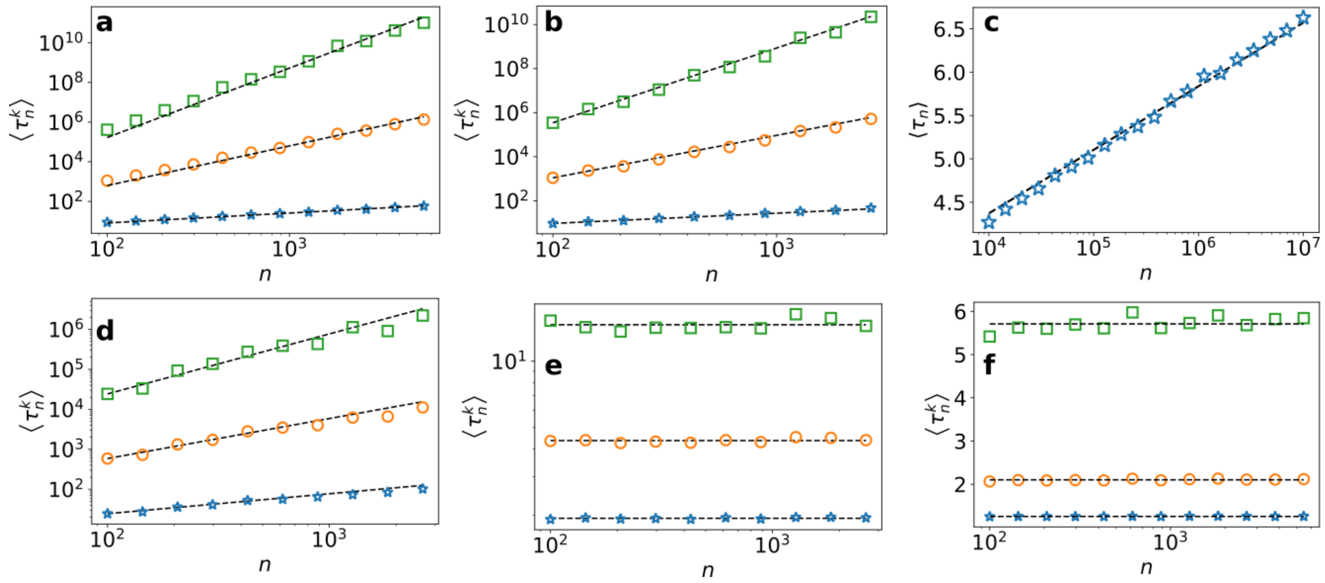

Supplementary Figure 8. **Moments of τ_n:** the predicted scaling behaviours are in black dashed line. We show the examples of **a** $1d$ Lévy flights of parameter $\alpha = 1.5$, **b** Sierpinski gasket nearest neighbour RW, **d** $2d$ lattice nearest neighbour RW, **e** $4d$ lattice nearest neighbour RW, **f** $1d$ Lévy flights of parameter $\alpha = 0.5$. **c** is the first moment of τ_n for the $2d$ lattice compared to $\frac{1}{\pi}\ln(n)$. For all figures except **d**, we represent the moments $\langle \tau_n^k \rangle$ for $k = 1$ (blue stars), $k = 2$ (orange circles) and $k = 3$ (green squares). For **d**, blue stars are related to $k = 2$, orange circles to $k = 3$, and green squares to $k = 4$.

S4. NUMERICAL SIMULATIONS

A. Recurrent random walks

The recurrent case is illustrated by Lévy flights in $1d$ with $\alpha \in]1,2[$ and by random walks on the Sierpinski gasket (fractal dimension $d_\mathrm{f} = \log 3/\log 2$ and walk dimension $d_\mathrm{w} = \log 5/\log 2$), the T-tree (Supplementary Figure 9**a**) with $d_\mathrm{f} = \log 3/\log 2$ and $d_\mathrm{w} = \log 6/\log 2$), and $2d$ critical percolation clusters ($d_\mathrm{f} = 91/48$ and $d_\mathrm{w} \approx 2.878$), using the algorithms described in Ref. [S16]. The Sierpinski gasket in our simulations is unbounded, while the T-tree has 9 generations. For these two examples, all walks start at the central site (inset to Fig. 3**b** of the main text for the Sierpinski gasket and Supplementary Figure 9**a** for the T-tree). The critical percolation cluster was constructed from a 1000×1000 periodic square lattice, from which half of the bonds were randomly removed and then the largest cluster was selected. Simulations were performed on the 31 clusters whose size is closest to the median size (out of an ensemble of 1000 clusters), with the starting site chosen randomly. Lévy flights are defined as follows: a jump length is drawn from the distribution $p(s) = 1/[2\zeta(1+\alpha)|s|^{1+\alpha}]$ for $s = \pm 1, \pm 2, \ldots$. Each jump takes one time unit. Intermediate sites between the starting and final sites of each jump are not visited. All these examples support the properties of the distribution $F_n(\tau)$ for $\mu < 1$ summarized in Table I of the main text, see also Fig. 3 of the main text and Supplementary Figure 9**a** for the T-tree.

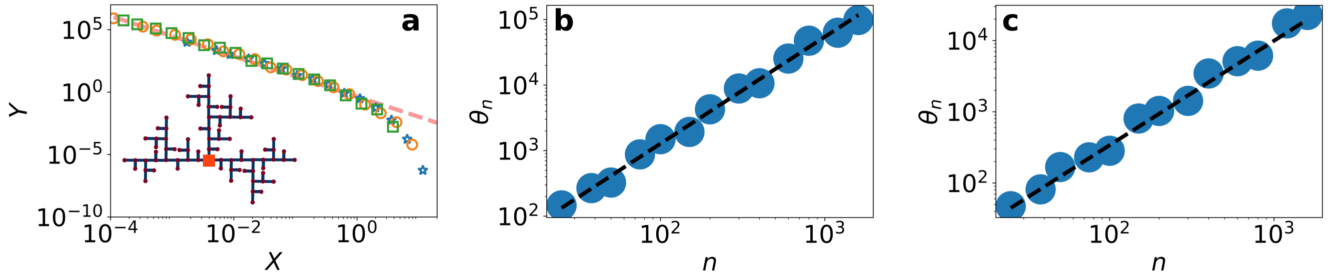

Supplementary Figure 9. **Recurrent random walks ($\mu < 1$): a** Universal distribution of the time between visits to new sites for a 9-generation T-tree for nearest-neighbor random walks. Plotted are simulation results for the scaled distribution $Y \equiv \theta_n^{1+\mu} F_n(\tau)$ versus $X \equiv \tau/\theta_n$ for $n = 100$, 500, and 1000 (respectively blue stars, orange circles, and green squares), where θ_n is the decay time in the exponential tail: $F_n(\tau) \sim \exp(-\tau/\theta_n)$. The dashed line indicates the algebraic decay $A(\mu)t^{-1-\mu}$ (see section S3.A.*4* for the definition of $A(\mu)$). The inset shows a 4-generation T-tree. **b** θ_n as a function of n for the T-tree compared to $n^{1/\mu}$ in black dashed line ($\mu = \frac{\ln 3}{\ln 6}$). **c** θ_n as a function of n for the Sierpinski gasket compared to $n^{1/\mu}$ in black dashed line ($\mu = \frac{\ln 3}{\ln 5}$).

In Supplementary Figure 9**b** and **c**, we show that θ_n the decay rate of the exponential regime indeed scales as $n^{1/\mu}$ in the examples of the T-tree and the Sierpinski gasket. We emphasize that the scaling of the moments shown in Supplementary Figure 8 and the scaling form of the distribution shown in Fig. 3 of the main text with θ_n are another direct demonstration of the existence of a single time scale $t_n = T_n = n^{1/\mu}$.

B. Exact enumeration technique

Evaluating the large time statistics $F_n(\tau)$ of τ_n, which can be of order $< 10^{-10}$ (see Fig. 3 of the main text), cannot be done by direct sampling of the event $\{\tau_n = t\}$, both for the recurrent and the transient cases. To overcome this problem, we rely on an exact enumeration technique adapted from Ref. [S17], which consists in evaluating the exit time statistics of the visited domain without generating the exiting trajectory but only the visited domain. The transient case also requires the Wang-Landau procedure [S18, S19] and importance sampling [S20], as described in section S4 C, because rare configurations of visited domains take part in the time statistics, contrary to the recurrent case (see Sec. S3 B).

We now explain our method in detail. Consider the situation when n distinct sites have been visited. We start from the last visited site. We are interested in the first exit time of the visited domain. We define $\mathbf{r}_n$ as the vector whose entries are given by the n distinct sites successively visited. Then we define $\mathbb{P}(\tau_n = t|\mathbf{r}_n)$ as the first exit-time probability of the RW at time t from the list of sites $\mathbf{r}_n$. We build a transition matrix $M(\mathbf{r}_n)$ from $\mathbf{r}_n$ based on the

single-step transition probability from the ith visited site to the jth visited site, $p(i \to j)$,

$$M(\mathbf{r}_n)_{j,i} \equiv p\left(i \to j\right) \ . \tag{56}$$

Next, we define v as the vector of length n with entries 0 except for the nth one which is 1 ($v_{i_0} = \delta_{i_0,n}$). As a consequence of the definition (56), and taking $t \geq 0$, we have that

$$\begin{aligned}\sum_i \left(M(\mathbf{r}_n)^t \cdot v\right)_i &= \sum_{i_0,\ldots,i_{t-1},i} M(\mathbf{r}_n)_{i,i_{t-1}} \ldots M(\mathbf{r}_n)_{i_1,i_0} v_{i_0} \\ &= \sum_{i_1,\ldots,i_{t-1},i} p(n \to i_1) p(i_1 \to i_2) \ldots p(i_{t-1} \to i) \ . \end{aligned} \tag{57}$$

We note that $p(n \to i_1)p(i_1 \to i_2)\ldots p(i_{t-1} \to i)$ is the probability to be successively at the i_1^{th}, ..., i_{t-1}^{th}, i^{th} visited site when starting from the nth visited site. Thus Eq. (57) expresses the exact enumeration of all of the trajectories of length t going through visited sites *only*, starting from the last visited site, by summing over all the values of i_1, ..., i_{t-1}, i. As a consequence, Eq. (57) is simply the probability of exiting the visited domain at a time larger or equal to $t+1$, $\mathbb{P}(\tau_n \geq t+1|\mathbf{r}_n)$. This results in

$$\mathbb{P}\left(\tau_n = t|\mathbf{r}_n\right) = \sum_{i=1}^{n} \left(\left(M(\mathbf{r}_n)^{t-1} - M(\mathbf{r}_n)^t\right) \cdot v\right)_i \ . \tag{58}$$

Now we use the following relation

$$\mathbb{P}\left(\tau_n = t\right) = \sum_{\mathbf{r}_n} \mathbb{P}\left(\tau_n = t|\mathbf{r}_n\right) \widetilde{\Pi}\left(\mathbf{r}_n\right) \ , \tag{59}$$

where $\widetilde{\Pi}(\mathbf{r}_n)$ is the generation probability to obtain the visited domain $\mathbf{r}_n$ of size n. Thus, to compute $\mathbb{P}\left(\tau_n = t\right)$, we are left with the sampling of $\mathbf{r}_n$, and the evaluation of $\mathbb{P}\left(\tau_n = t|\mathbf{r}_n\right)$ by Eq. (58). For recurrent and marginal RWs, $\mathbf{r}_n$ is sampled simply by drawing successive steps until n sites have been visited. For persistent random walks on hypercubic lattices, $M(\mathbf{r}_n)$ is now a $2dn \times 2dn$ matrix, where one adds to the description of a state its previous direction (there are $2d$ different directions).

C. Monte Carlo simulations of transient RWs

As explained in Sec. S4 B, for the transient RWs, we need, in addition to the exact enumeration, to sample the rare states of large trap-free regions. To do so, we use the technique of importance sampling [S20] and the method developed by Wang and Landau [S18, S19].

We focus on nearest-neighbor RWs on hypercubic lattices. Here $\boldsymbol{\sigma}_n = \boldsymbol{\sigma}$ (n implicit) is defined by the list of the $M_{\boldsymbol{\sigma}}$ successive steps σ_1, ..., $\sigma_{M_{\boldsymbol{\sigma}}}$ performed by the RW before having visited n distinct sites. This additional information is required for the Monte Carlo algorithm, as now the new generation probability $\Pi(\boldsymbol{\sigma})$ is given by

$$\Pi(\boldsymbol{\sigma}) = p(\sigma_1) \ldots p(\sigma_{M_{\boldsymbol{\sigma}}}) \ . \tag{60}$$

One can easily go from $\boldsymbol{\sigma}$ to $\mathbf{r}_n$ using that $\mathbf{r}_n$ can be obtained from the list $\left[0, \sigma_1, \sigma_1 + \sigma_2, \ldots, \sum_{i \geq M_{\boldsymbol{\sigma}}} \sigma_i\right]$ by removing sites whenever they reappear, keeping the first occurrence at the same position. Thus, we define $\mathbb{P}\left(\tau_n = t|\boldsymbol{\sigma}\right)$ as $\mathbb{P}\left(\tau_n = t|\mathbf{r}_n\right)$ with the list $\mathbf{r}_n$ related to $\boldsymbol{\sigma}$.

1. Importance sampling: general idea

The idea is to bias the generation probability $\Pi(\boldsymbol{\sigma})$ towards states which contribute the most to the statistics we want to sample. Here, the statistics to sample is $\mathbb{P}\left(\tau_n = t\right)$, which can be seen as the average of the observable $\mathbb{P}\left(\tau_n = t|\boldsymbol{\sigma}\right)$ over the generation probability as

$$\mathbb{P}\left(\tau_n = t\right) = \sum_{\boldsymbol{\sigma}} \mathbb{P}\left(\tau_n = t|\boldsymbol{\sigma}\right) \Pi(\boldsymbol{\sigma}) \ . \tag{61}$$

We associate to every state $\boldsymbol{\sigma}$ an energy $E(\boldsymbol{\sigma})$, such that rare states are associated to low energies. We now bias the generation probability $\Pi(\boldsymbol{\sigma})$ in the following way,

$$\mathbb{P}\left(\tau_n = t\right) = \sum_{\boldsymbol{\sigma}} \mathbb{P}\left(\tau_n = t|\boldsymbol{\sigma}\right) \Pi(\boldsymbol{\sigma}) = \sum_{\boldsymbol{\sigma}} \mathbb{P}\left(\tau_n = t|\boldsymbol{\sigma}\right) \frac{g\left(E(\boldsymbol{\sigma})\right)}{g\left(E(\boldsymbol{\sigma})\right)} \Pi(\boldsymbol{\sigma}) \ , \tag{62}$$

where g is the bias that we want to introduce. This allows us to define the biased probability distribution $\Pi_g(\boldsymbol{\sigma}) = \Pi(\boldsymbol{\sigma}) g\left(E(\boldsymbol{\sigma})\right)^{-1} Z_g^{-1}$, where Z_g is a normalization constant. Thus we have

$$\mathbb{P}\left(\tau_n = t\right) = \sum_{\boldsymbol{\sigma}} \mathbb{P}\left(\tau_n = t|\boldsymbol{\sigma}\right) g\left(E(\boldsymbol{\sigma})\right) Z_g \Pi_g(\boldsymbol{\sigma}) = \frac{\left\langle \mathbb{P}\left(\tau_n = t|\boldsymbol{\sigma}\right) g\left(E(\boldsymbol{\sigma})\right)\right\rangle_g}{\left\langle g\left(E(\boldsymbol{\sigma})\right)\right\rangle_g}, \tag{63}$$

where we have used $Z_g^{-1} = \left\langle g\left(E(\boldsymbol{\sigma})\right)\right\rangle_g$, since $\sum_t \mathbb{P}\left(\tau_n = t|\boldsymbol{\sigma}\right) = 1$, and noting $\langle \ldots \rangle_g$ the average with respect to the distribution Π_g. The terms $\mathbb{P}\left(\tau_n = t|\boldsymbol{\sigma}\right)$ are computed as described in section S4 B. If the bias is wisely chosen (by enhancing the probability of low-energy states) the error in the estimation of the average of $g\left(E(\boldsymbol{\sigma})\right) \mathbb{P}\left(\tau_n = t|\boldsymbol{\sigma}\right) Z_g^{-1}$, drawn from $\Pi_g(\boldsymbol{\sigma})$, will be smaller than the error that comes from $\mathbb{P}\left(\tau_n = t|\boldsymbol{\sigma}\right)$, drawn from $\Pi(\boldsymbol{\sigma})$. Thus, this technique allows us to sample statistics that are usually not observable, because of the error made by averaging is much bigger than the estimated value itself.

2. Choice of the energy

In this section, we choose a definition of the energy $E(\boldsymbol{\sigma})$, and give the reasons that motivate this choice. We start from Eq. (25): the (average) return time to the boundary (where traps are located) is inversely proportional to the domain size, *i.e.,* the perimeter P of the visited domain of fixed volume n. Thus, small perimeters P correspond to the large exit times statistics we want to sample. Additionally, defining L as the number of pairs (i, j) of visited sites at distance 1 (with no regards on the order), we have the following relation between P and L

$$P + 2L = 2dn \tag{64}$$

for a d-dimensional hypercubic lattice. Indeed, each of the n sites has $2d$ nearest neighbors (visited or not). Thus, one needs to maximize L in order to minimize P, which in turn gives access to the exit-time statistics at long times. These considerations result in the following choice for the energy of a state $\boldsymbol{\sigma}$:

$$E(\boldsymbol{\sigma}) \equiv -L \ . \tag{65}$$

3. The Monte Carlo moves: example

One needs to define the steps that can be made between successive states $\boldsymbol{\sigma}$. Similar to [S21], we developed the following algorithm, based on successive step exchanges:

We illustrate the method for the example in Supplementary Figure 10. We start from a state $\boldsymbol{\sigma}_9$ of 10 steps (red arrows). By drawing uniformly between 1 and 10, we obtain 7: we now exchange the 7th and 8th steps indicated by the green arrow. We obtain a new list of steps where $7'$ is the previous 8th step, $8'$ is the previous 7th step, and the rest of the trajectory is the same. However, this new list of steps gives one less visited site (green cross). Thus we add a new step 11 (blue arrow) randomly drawn from the transition probability (here, because we make a nearest-neighbor RW, we choose uniformly between the 4 directions). This new step leads to the visit of a new site. Thus we end up with a new list $\boldsymbol{\sigma}'_9$, which consists of 11 RW steps that correspond to 9 distinct visited sites.

This algorithm results in the following probability of proposing a given state $\boldsymbol{\sigma'} \equiv \boldsymbol{b}$ ($M_{\boldsymbol{b}}$ steps) starting from state $\boldsymbol{\sigma} \equiv \boldsymbol{a}$ ($M_{\boldsymbol{a}}$ steps),

$$p_{\text{prop}}(\boldsymbol{a} \to \boldsymbol{b}) = \begin{cases} \dfrac{p(b_{M_{\boldsymbol{a}}+1}) \ldots p(b_{M_{\boldsymbol{b}}})}{M_{\boldsymbol{a}}} & \text{if } M_{\boldsymbol{b}} > M_{\boldsymbol{a}}, \\ \dfrac{1}{M_{\boldsymbol{a}}} & \text{otherwise,} \end{cases} \tag{66}$$

given that $\boldsymbol{b}$ can be obtained from $\boldsymbol{a}$ with the successive steps exchange algorithm. The factor $1/M_{\boldsymbol{a}}$ corresponds to the choice of the step we change (either exchange of step i and $i+1$ or change of the last one if $i = M_{\boldsymbol{a}}$), $p(b_i)$ being

Algorithm 1: Monte-Carlo move algorithm, MC($\boldsymbol{\sigma}$)

Given a state $\boldsymbol{\sigma}$ of steps σ_1 ..., $\sigma_{M_{\boldsymbol{\sigma}}}$ of n distinct visited sites ;
Choose uniformly one index i between 1 and $M_{\boldsymbol{\sigma}}$ (included) ;
if $i < M_{\boldsymbol{\sigma}}$ **then**
  | Exchange steps σ_i and σ_{i+1}
else
  | Draw a new step $\sigma_{M_{\boldsymbol{\sigma}}+1}$ from p ;
  | Add it to the list of steps ;
  | Exchange steps $\sigma_{M_{\boldsymbol{\sigma}}}$ and $\sigma_{M_{\boldsymbol{\sigma}}+1}$;
end
while *the number of visited sites* $< n$ **do**
  | Add steps drawn from the transition probability p to the end of the list of steps ;
end
while *the number of visited sites* $> n$ *or the last step points to a site that has been visited several times* **do**
  | Remove the last step ;
end
Return the new state $\boldsymbol{\sigma}'$

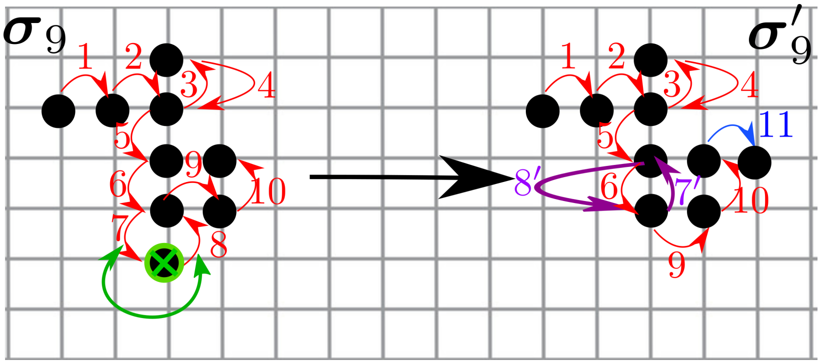

Supplementary Figure 10. **Example of Monte Carlo move.** Steps are red arrows, visited sites are in black. The suppressed visited site is green crossed. The green arrow points to the the two steps (in purple) that have been exchanged, and in blue the added step to keep the number of distinct sites visited fixed.

the transition probability to make the jump b_i in one time step ($p(b_i) = 1/(2d)$ for the nearest-neighbor RW on the hypercubic lattice). The other factors correspond to the right choice of successive added steps which lead to $\boldsymbol{b}$.

Now that we have our algorithm to move from one state to another, we return to the problem of sampling Π_g. To this end, we consider the detailed balance condition

$$\pi(\boldsymbol{a} \to \boldsymbol{b})\Pi_g(\boldsymbol{a}) = \pi(\boldsymbol{b} \to \boldsymbol{a})\Pi_g(\boldsymbol{b}) , \qquad \text{with} \qquad \pi(\boldsymbol{a} \to \boldsymbol{b}) = p_{\text{prop}}(\boldsymbol{a} \to \boldsymbol{b}) p_{\text{acc}}(\boldsymbol{a} \to \boldsymbol{b}) , \tag{67}$$

where $p_{\text{acc}}(\boldsymbol{a} \to \boldsymbol{b})$ is the probability to accept the Monte Carlo move. We use a classical result from Markov chain theory: if we use an algorithm able to draw transitions between states with probability $\pi(\boldsymbol{a} \to \boldsymbol{b})$ following the detailed balance condition of Eq. (67), then the states obtained from the algorithm are distributed according to Π_g in the steady state. Now, using that

$$\Pi_g(\boldsymbol{a}) = p(a_1)\dots p(a_{M_{\boldsymbol{a}}}) g(E(\boldsymbol{a}))^{-1} Z_g^{-1} , \tag{68}$$

the detailed balance condition becomes using Eqs. (66) and (67), and taking without loss of generality $M_{\boldsymbol{a}} \leq M_{\boldsymbol{b}}$,

$$p_{\text{acc}}(\boldsymbol{a} \to \boldsymbol{b}) g(E(\boldsymbol{a}))^{-1} Z_g^{-1} \frac{1}{M_{\boldsymbol{a}}} p(a_1)\dots p(a_{M_{\boldsymbol{a}}}) p(b_{M_{\boldsymbol{a}}+1})\dots p(b_{M_{\boldsymbol{b}}}) = p_{\text{acc}}(\boldsymbol{b} \to \ \boldsymbol{a}) g(E(\boldsymbol{b}))^{-1} Z_g^{-1} \frac{1}{M_{\boldsymbol{b}}} p(b_1)\dots p(b_{M_{\boldsymbol{b}}}) \tag{69}$$

in the case for which the algorithm permits a move from $\boldsymbol{a}$ to $\boldsymbol{b}$ in a single Monte Carlo step. Thus we take the following acceptance rate using the Metropolis-Hastings prescription [S22]

$$p_{\text{acc}}(\boldsymbol{a} \to \boldsymbol{b}) = \min\left(1, \frac{M_{\boldsymbol{a}} g(E(\boldsymbol{a}))}{M_{\boldsymbol{b}} g(E(\boldsymbol{b}))}\right) . \tag{70}$$

4. Choice of the bias: Wang-Landau

To use the procedure described in the previous subsection we have to make a choice of the bias g. We follow the Wang-Landau prescription to sample uniformly the energy levels, biasing them via the approximation of the energy distribution obtained by the algorithm [S18, S19]. To obtain $g(E)$, the idea is to explore the energy landscape $E(\boldsymbol{\sigma})$ via the Monte-Carlo moves described in Sec. S4 C. Every time we observe an energy E, we update our knowledge of the energy density $g(E)$ and use Eq. (70) to bias the random walk on the energy landscape towards rare states (low values of $g(E)$). This has the effect of flattening the observed energy landscape, as can be seen by computing the energy histogram $H(E)$. We end the algorithm when we observe each energy level with similar probability, *i.e.*, when the energy landscape is flat enough.

A realization of this algorithm is the following:

Algorithm 2: Wang-Landau algorithm to obtain the energy density $\mathcal{N}(E)$

Given a state $\boldsymbol{\sigma}$, a uniform distribution $g(E)$, a factor $f = \exp(1)$ and an energy histogram $H(E)$ with entries 0 **while** $f > \exp(10^{-8})$ **do**
- Draw $\boldsymbol{\sigma}'$ from $\mathrm{MC}(\boldsymbol{\sigma})$;
- Replace $\boldsymbol{\sigma}$ by $\boldsymbol{\sigma}'$ with probability $p_{\mathrm{acc}}(\boldsymbol{\sigma} \to \boldsymbol{\sigma}')$ (see Eq. (70)) ;
- Multiply $g(E(\boldsymbol{\sigma}))$ by f, $g(E(\boldsymbol{\sigma})) \to f \times g(E(\boldsymbol{\sigma}))$;
- Increase the energy histogram $H(E(\boldsymbol{\sigma}))$ by 1, $H(E(\boldsymbol{\sigma})) \to 1 + H(E(\boldsymbol{\sigma}))$;
- **if** $\sum_E H(E) > 1000$ *and each value of* $H(E)$ *is larger than* $0.9 \frac{1}{N_E} \sum_E H(E)$ *(*N_E *the number of energy levels)* **then**
  - Reset all the values of the histogram $H(E)$ to 0 ;
  - Replace the value of f by $\sqrt{f}$;
- **end**

end

Return $g(E)/\sum_E g(E)$ which approximates the energy density $\mathcal{N}(E)$

5. Results of the Monte Carlo simulations

We apply the method developed above to nearest-neighbor RWs on the cubic lattice and obtained Fig. 4**c** of the main text. The results for hypercubic lattices in dimensions $d = 4$, 5 and 6 are displayed in Supplementary Figure 11. Using appropriately scaled units, we verify the stretched exponential and the exponential long-time regimes.

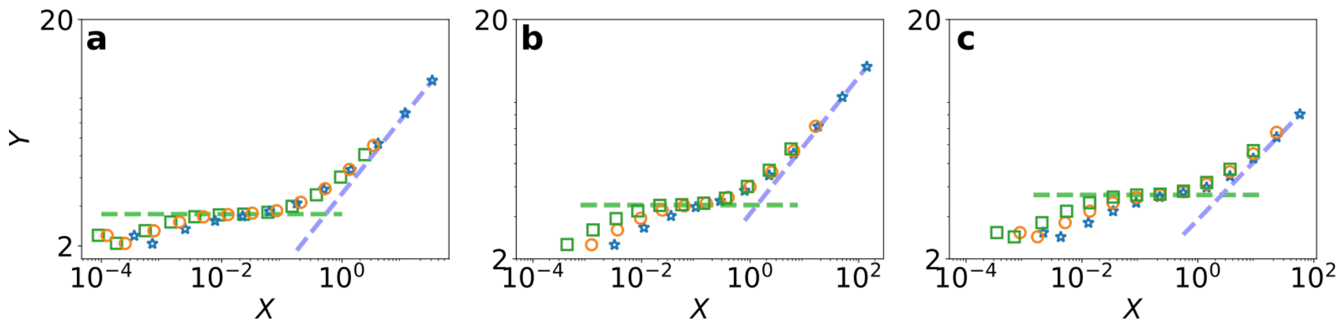

Supplementary Figure 11. **Transient nearest neighbour RWs** ($\mu > 1$) **on hypercubic lattice.** The scaled distribution $Y \equiv \left[-(\ln t_n)^2 F_n(\tau)\right] / (\tau/t_n)^{\mu/(1+\mu)}$ versus $X \equiv \tau/T_n$ for nearest-neighbor RWs in: **a** $d = 4$ (for $n = 200$, 400, and 500, blue stars, orange circles, and green squares, respectively), **b** $d = 5$, and **c** $d = 6$ (for $n = 100$, 200, and 400, blue stars, orange squares, and green squares, respectively). The early-time plateau illustrates the stretched exponential regime (horizontal green dashed line) and the blue dashed line, proportional to $X^{1/(1+\mu)}$, corresponds to the exponential regime.

6. *Stretched exponential without the Monte Carlo method*

Even for random walks where the Monte Carlo technique of Sec S4.C.*5* does not apply, we can still observe the beginning of the stretched exponential law using the exact enumeration method. Here we show in Supplementary Figure 12 two other transient walks, the $1d$ Lévy flight of parameter $\alpha = 0.5$ and the $3d$ Lévy flight of parameter $\alpha = 1.2$. Both display the stretched exponential behaviour as expected.

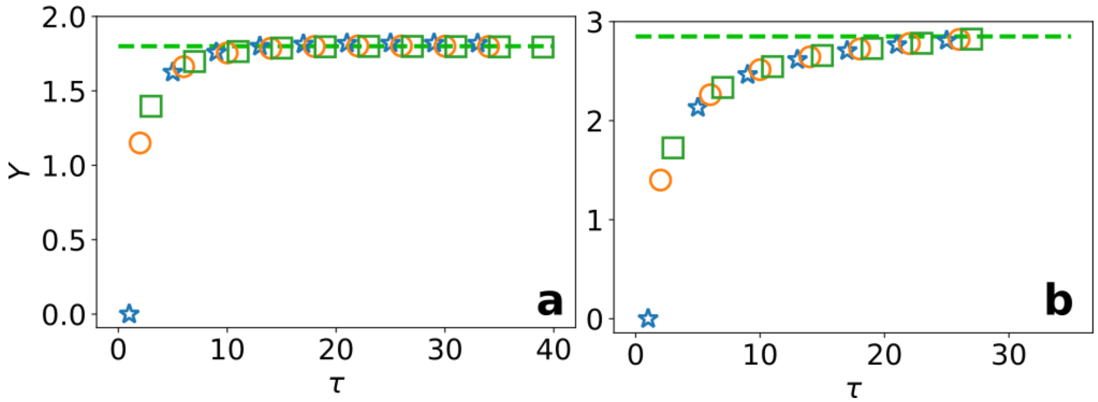

Supplementary Figure 12. **Transient Lévy flights:** $Y = \frac{-\ln F_n(\tau)}{\tau^{\mu/(1+\mu)}}$ for Lévy flights of parameter **a** $\alpha = 0.5$ in $1d$ **b** $\alpha = 1.2$ in $3d$ for $n = 400$, 800, and 1600 (blue stars, orange circles, and green squares, respectively). Both are compared to a constant value (dashed line).

S5. EXTENSIONS

A. General observables

1. *Definition of bulk and boundary observables*

A more complete characterization of the visitation statistics of random walks is provided by introducing two classes of more general visitation observables. A visitation observable is called *bulk* if only visited sites are involved in its characterization. Otherwise it is a *boundary* (or surface) observable (see [S14, S23] for precise definitions). According to these definitions, the number $L(t)$ of links/adjacent visited sites is a bulk observable (illustrated in Supplementary Figure 13), while the perimeter $P(t)$ of the visited domain and the number $I(t)$ of islands are boundary observables.

2. *Dynamics of bulk observables*

We argue that the dynamics of bulk variables is the same as that for the number of new sites visited. For the example of the number of new links $L(t)$ visited, both for a nearest-neighbor RW on a square lattice and a $1d$ Lévy flight with $\alpha = 1$, we check in Supplementary Figure 13**c** and **d** that the distribution of times τ_l between successive creation of links is similar to the distribution of times τ_n between successive visits to new sites.

3. *Dynamics of boundary observables*

We now derive the exponent ϕ of the algebraic decay $F_\Sigma(\tau) \sim \tau^{-1-\phi}$ for recurrent and marginal RWs for a boundary observable Σ, as defined in the main text. This derivation follows the spirit of Section S3.A.*3*. The first step consists in realizing that the finite-size cutoff time t_Σ in the dynamics of a surface observable Σ is the same as the cutoff time $t_n \sim n^{1/\mu}$ for the number n of distinct sites visited. Next, we use the known result (see [S14, S23]) that for a boundary observable Σ,

$$\langle \Sigma(t) \rangle \sim t^{2\mu-1} \,. \tag{71}$$

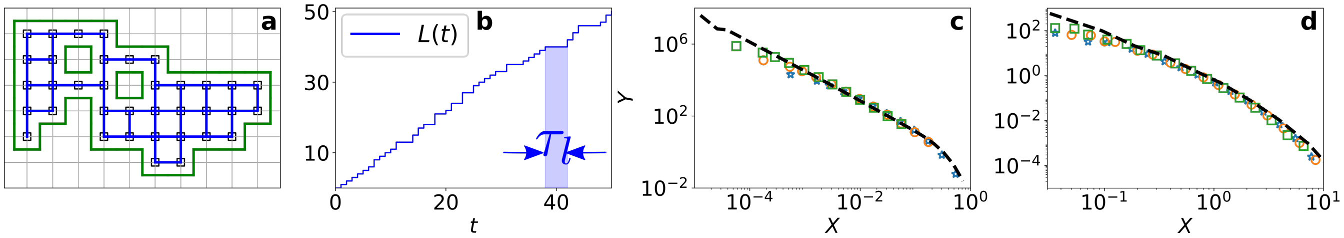

Supplementary Figure 13. **Extension to general "bulk" observables for recurrent and marginal RWs.** **a** An example of bulk observable: the number of links in the support of the walk. The blue lines are the links in the visited domain. **b** Time dependence of the number of links $L(t)$. The shaded area represents the waiting time τ_l between two increments of $L(t)$. **c** and **d** Comparison between the rescaled distributions of τ_l, $Y \equiv t_l^{1+\mu} F_l(\tau/t_l)$ versus $X = \tau/t_l$ (color) and the rescaled distributions of τ_n, $Y \equiv t_n^{1+\mu} F_n(\tau/t_n)$ versus $X \equiv \tau/t_n$ (black) for **c** nearest-neighbor RWs on the square lattice and **d** Lévy flights of parameter $\alpha = 1/\mu = \ln 6/\ln 3$. In these plots, l takes the values 100, 200 and 400 (blue stars, orange circles, green squares), while $n = 1000$ (black dashed line). Here $t_l = l^{1/\mu}$ if $\mu < 1$, $t_l = \sqrt{l}$ if $\mu = 1$, and similarly for t_n.

Using the scaling of the number of distinct sites visited with time, $t \sim n^{1/\mu}$, we obtain the cutoff time in terms of value of Σ instead of n:

$$t_\Sigma \sim \Sigma^{1/(2\mu-1)} \,. \tag{72}$$

We are now in a position to identify two expressions of the first moment. First, relying on its definition and the algebraic behavior $F_\Sigma(\tau) \propto \tau^{-1-\phi}$, we have

$$\langle \tau_\Sigma \rangle \propto \int_1^{t_\Sigma} \frac{t}{t^{1+\phi}}\,\mathrm{d}t \propto t_\Sigma^{1-\phi} = \Sigma^{(1-\phi)/(2\mu-1)} \,. \tag{73}$$

Second, we use that

$$\frac{\mathrm{d}\langle \Sigma \rangle}{\mathrm{d}t} \sim \frac{1}{\langle \tau_\Sigma \rangle} \sim t^{2\mu-2} \,, \tag{74}$$

which leads, with the help of Eq. (71), to

$$\langle \tau_\Sigma \rangle \sim \Sigma^{(2-2\mu)/(2\mu-1)}. \tag{75}$$

Combining (73) and (75), and matching the exponents, we finally obtain

$$\phi = 2\mu - 1, \tag{76}$$

as given in the main text.

In the marginal case, we have [S14, S23]

$$\langle \Sigma(t) \rangle \sim \frac{t}{\ln^2 t}. \tag{77}$$

Using as previously in Eq. (74) that

$$\frac{\mathrm{d}\,\langle \Sigma(t) \rangle}{\mathrm{d}t} \sim \frac{1}{\left\langle \tau_{\langle \Sigma(t) \rangle} \right\rangle} \,, \tag{78}$$

we find

$$\langle \tau_\Sigma \rangle \sim \ln^2 t. \tag{79}$$

The definition of $\langle \tau_\Sigma \rangle$ in terms of F_Σ finally yields

$$F_\Sigma(\tau) \propto \frac{\ln \tau}{\tau^2} \,, \tag{80}$$

as given in the main text. Equations (76) and (80) are numerically confirmed in Fig. 4**b** and **c** in the main text.

B. Covariance of the number of distinct sites visited for recurrent $1d$ Lévy flights

1. *Derivation of the multiple time correlations*

One can generalize the result on the covariance $\mathrm{Cov}[N(t_1), N(t_2)]$, Eq. (6) of the main text, to

$$\langle (N(t_1) - \langle N(t_1)\rangle) \dots (N(t_k) - \langle N(t_k)\rangle)\rangle \propto t_1^{1/\alpha} \dots t_k^{1/\alpha} \, \frac{t_1}{t_k} \, . \tag{81}$$

To obtain this result, we start from (making the hypothesis that correlations between τ_k can be neglected)

$$\mathcal{L}\{\mathbb{P}(N(t_1) \geq n_1, \dots, N(t_k) \geq n_k)\} = \frac{1}{s_1 \dots s_k} \frac{h(0)}{h\big((s_1 + \dots + s_k) n_1^{1/\mu}\big)} \dots \frac{h(s_k n_{k-1}^{1/\mu})}{h(s_k n_k^{1/\mu})} \, , \tag{82}$$

where

$$h(s,n) \equiv h(s n^{1/\mu}) = \exp\left[\int_0^n \left(\mathcal{L}\{\mathbb{P}(\tau_k = t)\} - 1\right) \mathrm{d}k\right] \, . \tag{83}$$

This is an extension of the calculation performed in [S24]. By supposing that the correlations between τ_k can be neglected, one obtains a simple formula for the distribution of the number of distinct sites visited at k different times as a product of functionals of the exit time distribution. We consider the limit $t_1 \ll \dots \ll t_k$, i.e., $s_1 \gg \dots \gg s_k$ and $n_i s_i^{\mu} = a_i$ fixed. For $k = 2$, we notice that

$$\begin{aligned}
&\mathcal{L}\{\mathbb{P}(N(t_1) \geq n_1, N(t_2) \geq n_2)\} \\
&\approx \frac{1}{s_1 s_2} \frac{h(0)}{h(a_1^{1/\mu})} \frac{h(0)}{h(a_2^{1/\mu})} + \frac{s_2}{s_1} \frac{1}{s_1 s_2} \left(\frac{h(0)}{h(a_1^{1/\mu})} \frac{h'(0) a_1^{1/\mu}}{h(a_2^{1/\mu})} - \frac{h(0) h'(a_1^{1/\mu}) a_1^{1/\mu}}{h(a_1^{1/\mu})^2} \frac{h(0)}{h(a_2^{1/\mu})} \right) \\
&= \mathcal{L}\{\mathbb{P}(N(t_1) \geq n_1)\} \mathcal{L}\{\mathbb{P}(N(t_2) \geq n_2)\} + a_1^{1/\mu} \frac{h'(0) h(a_1^{1/\mu}) - h'(a_1^{1/\mu})}{s_1 s_2 h(a_2^{1/\mu}) h(a_1^{1/\mu})^2} \frac{s_2}{s_1} \, .
\end{aligned} \tag{84}$$

Once integrated over n_1 and n_2, we obtain the covariance's scaling mentioned in the main text,

$$\mathrm{Cov}\left[N(t_1), N(t_2)\right] \propto \frac{t_1}{t_2} t_1^{\mu} t_2^{\mu} \, . \tag{85}$$

For $k = 3$, we start from

$$\begin{aligned}
&\langle (N(t_1) - \langle N(t_1)\rangle)(N(t_2) - \langle N(t_2)\rangle)(N(t_3) - \langle N(t_3)\rangle)\rangle \\
&= \langle N(t_1) N(t_2) N(t_3)\rangle - \langle N(t_1)\rangle \langle N(t_2) N(t_3)\rangle - \langle N(t_2)\rangle \langle N(t_1) N(t_3)\rangle \\
&\quad - \langle N(t_3)\rangle \langle N(t_1) N(t_2)\rangle + 2 \langle N(t_3)\rangle \langle N(t_1)\rangle \langle N(t_2)\rangle \, .
\end{aligned} \tag{86}$$

Thus to compute the correlation function, we are left with the comparison of the three-time distribution which results in $\langle N(t_1) N(t_2) N(t_3)\rangle$ and the sum of distributions giving rise to the other moments.

$$\begin{aligned}
&\mathcal{L}\{\mathbb{P}(N(t_1) \geq n_1, N(t_2) \geq n_2, N(t_3) \geq n_3)\} = \mathcal{L}\{\mathbb{P}(N(t_1) \geq n_1)\} \mathcal{L}\{\mathbb{P}(N(t_2) \geq n_2, N(t_3) \geq n_3)\} \\
&+ \mathcal{L}\{\mathbb{P}(N(t_2) \geq n_2)\} \mathcal{L}\{\mathbb{P}(N(t_1) \geq n_1, N(t_3) \geq n_3) + \mathcal{L}\{\mathbb{P}(N(t_3) \geq n_3)\} \mathcal{L}\{\mathbb{P}(N(t_1) \geq n_1, N(t_2) \geq n_2)\}\} \\
&- 2\mathcal{L}\{\mathbb{P}(N(t_1) \geq n_1)\} \mathcal{L}\{\mathbb{P}(N(t_2) \geq n_2)\} \mathcal{L}\{\mathbb{P}(N(t_3) \geq n_3)\} \\
&+ a_1^{1/\mu} a_2^{1/\mu} \frac{h'(0)^2 h(a_1^{1/\mu}) h(a_2^{1/\mu}) - h'(0) h'(a_1^{1/\mu}) h(a_2^{1/\mu}) - h'(0) h(a_1^{1/\mu}) h'(a_2^{1/\mu}) + h'(a_1^{1/\mu}) h'(a_2^{1/\mu})}{s_1 s_2 s_3 h(a_3^{1/\mu}) h(a_1^{1/\mu})^2 h(a_2^{1/\mu})^2} \frac{s_3}{s_1} \\
&+ o\left(\frac{s_3}{s_1}\right) \, .
\end{aligned} \tag{87}$$

By integrating this equation over n_1, n_2 and n_3 and using the Tauberian theorem we obtain the scaling of (81). Generalising the calculation to any number of times gives (81).

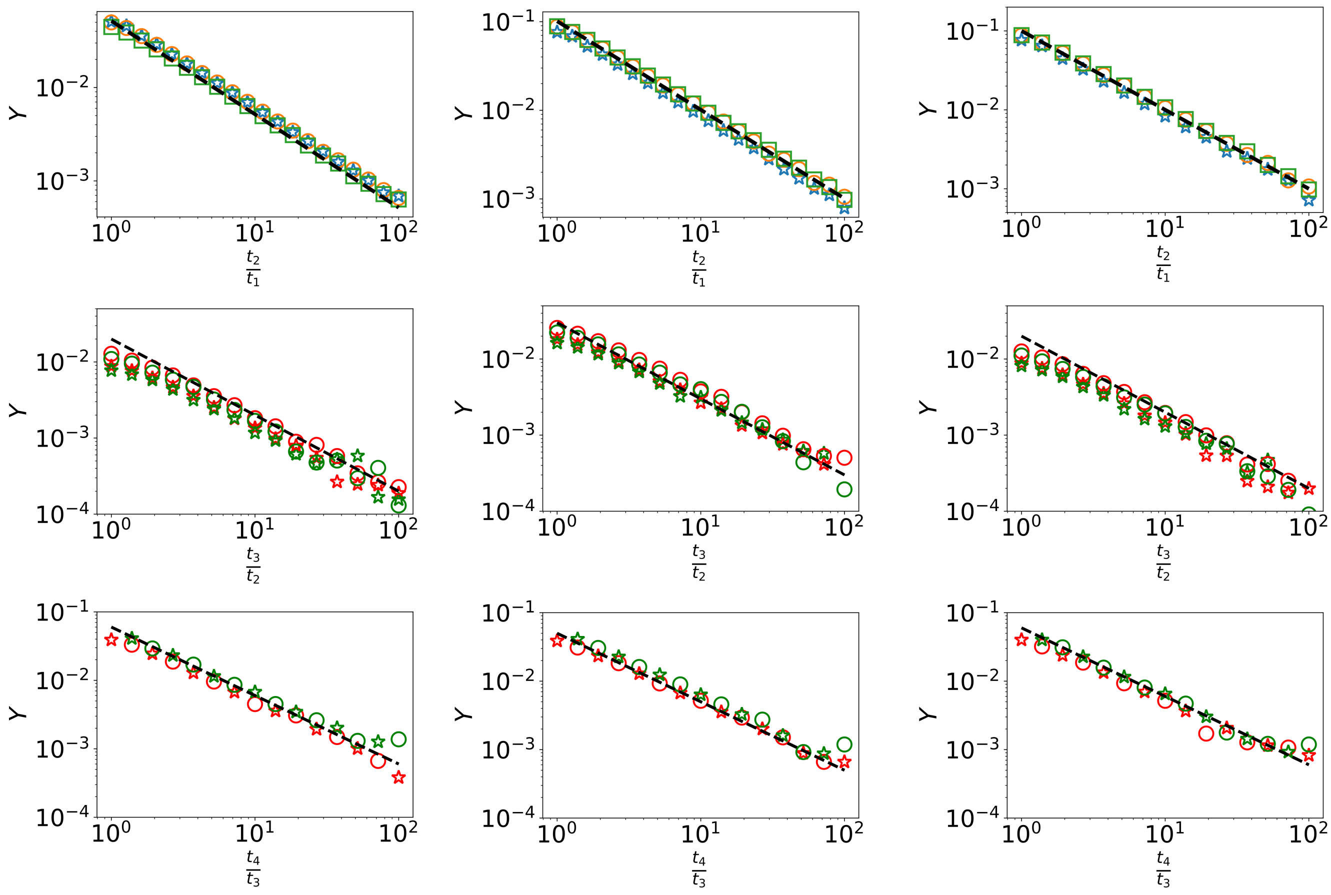

Supplementary Figure 14. **Multiple times correlation function of Lévy flights:** The parameter α takes the values $\alpha = 1.3$ (left column), $\alpha = 1.5$ (middle column) and $\alpha = 1.7$ (right column).
Upper panel: $Y = \langle((N(t_1) - \langle N(t_1)\rangle)(N(t_2) - \langle N(t_2)\rangle))\rangle / (\langle N(t_1)\rangle \langle N(t_2)\rangle)$, and we compare Y to the black dashed line proportional to $\frac{t_1}{t_2}$. Stars stand for $t_1 = 10$, circles for $t_1 = 100$ and squares for $t_1 = 1000$.
Middle panel: $Y = \langle((N(t_1) - \langle N(t_1)\rangle)(N(t_2) - \langle N(t_2)\rangle)(N(t_3) - \langle N(t_3)\rangle))\rangle / (\langle N(t_1)\rangle \langle N(t_2)\rangle \langle N(t_3)\rangle)\frac{t_2}{t_1}$, and we compare Y to the black dashed line proportional to $\frac{t_2}{t_3}$. Plots in red stand for $t_1 = 10$, in green $t_1 = 100$. Stars stand for $t_2 = 2t_1$ and circles for $t_2 = 4t_1$.
Lower panel: $Y = \langle((N(t_1) - \langle N(t_1)\rangle)(N(t_2) - \langle N(t_2)\rangle)(N(t_3) - \langle N(t_3)\rangle)(N(t_4) - \langle N(t_4)\rangle))\rangle / (\langle N(t_1)\rangle \langle N(t_2)\rangle \langle N(t_3)\rangle \langle N(t_4)\rangle)\frac{t_3}{t_1}$, and we compare Y to the black dashed line proportional to $\frac{t_3}{t_4}$. Red symbols stand for $t_1 = 10$ and green for $t_1 = 100$. Stars stand for $t_2 = 2t_1$ and circles for $t_2 = 4t_1$. In all three lower subplots we take $t_3 = 4t_2$.

2. *Criterion on τ_n correlations*

Our starting point is a relation between the number of distinct sites visited and the sum of τ_k,

$$\{N(t) \geq n\} = \left\{\sum_{k=1}^{n-1} \tau_k \leq t\right\} . \tag{88}$$

Then, using that the rescaled process $N(t)/t^\mu$ is asymptotically independent of t for recurrent random walks (rigorously proved in the mathematical literature in the case of recurrent Lévy flights [S25]), we have that $\sum_{k=1}^{n} \tau_k/n^{1/\mu}$ is independent of n because

$$\mathbb{P}(N(t)/t^\mu \geq 1) = \mathbb{P}\left(\frac{\sum_{k=1}^{t^\mu-1} \tau_k}{t} \leq 1\right) . \tag{89}$$

We note $\kappa_i\left(\sum_{k=1}^{n}\tau_k\right)=A_i n^{i/\mu}$, κ_i being the ith cumulant. We start from the relation between the number $N(t)$ of distinct sites visited and the τ_k and perform a cumulant expansion,

$$\begin{aligned}
&\mathcal{L}\{\mathbb{P}(N(t_1)\geq n_1, N(t_2)\geq n_2)\}\\
&=\sum_{t_1,t_2}\exp\left[-s_1t_1-s_2t_2\right]\mathbb{P}\left(\sum_{k=0}^{n_1-1}\tau_k\leq t_1,\ \sum_{k=0}^{n_2-1}\tau_k\leq t_2\right)\\
&=\frac{1}{s_1s_2}\mathbb{E}\left(\exp\left[-s_1\sum_{k=0}^{n_1-1}\tau_k-s_2\sum_{k=0}^{n_2-1}\tau_k\right]\right)\\
&=\frac{1}{s_1s_2}\exp\left[\sum_{i=1}^{\infty}\frac{1}{i!}\kappa_i\left(-s_1\sum_{k=0}^{n_1-1}\tau_k-s_2\sum_{k=0}^{n_2-1}\tau_k\right)\right]\ ,
\end{aligned}\tag{90}$$

which we compare to the product of single time distributions,

$$\mathcal{L}\{\mathbb{P}(N(t)\geq n)\}=\frac{1}{s}\exp\left[\sum_{i=1}^{\infty}\frac{1}{i!}\kappa_i\left(-s\sum_{k=0}^{n-1}\tau_k\right)\right]\equiv\frac{1}{s}H(sn^{1/\mu})\ .\tag{91}$$

Keeping only the first non-zero terms (which appear with the second cumulant), we obtain

$$\begin{aligned}
&\mathcal{L}\{\mathbb{P}(N(t_1)\geq n_1, N(t_2)\geq n_2)\}-\mathcal{L}\{\mathbb{P}(N(t_1)\geq n_1)\}\mathcal{L}\{\mathbb{P}(N(t_2)\geq n_2)\}\\
&\approx\mathcal{L}\{\mathbb{P}(N(t_1)\geq n_1)\}\mathcal{L}\{\mathbb{P}(N(t_2)\geq n_2)\}\left(\exp\left[s_1s_2A_2n_1^{2/\mu}+s_1s_2\mathrm{Cov}\left[\sum_{k=0}^{n_1-1}\tau_k,\sum_{k=n_1}^{n_2-1}\tau_k\right]\right]-1\right)\\
&\approx\mathcal{L}\{\mathbb{P}(N(t_1)\geq n_1)\}\mathcal{L}\{\mathbb{P}(N(t_2)\geq n_2)\}\left(s_1s_2A_2n_1^{2/\mu}+s_1s_2\mathrm{Cov}\left[\sum_{k=0}^{n_1-1}\tau_k,\sum_{k=n_1}^{n_2-1}\tau_k\right]\right)\ .
\end{aligned}\tag{92}$$

We are interested in the term which will dominate the covariance for values $t_1\ll t_2$ i.e. $s_1\gg s_2$. We integrate the first term over n_1 and n_2 to obtain the first term of the covariance,

$$\begin{aligned}
&\int\mathrm{d}n_1\mathrm{d}n_2\mathcal{L}\{\mathbb{P}(N(t_1)\geq n_1)\}\mathcal{L}\{\mathbb{P}(N(t_2)\geq n_2)\}s_1s_2A_2n_1^{2/\mu}\\
&\qquad\approx\frac{s_2}{s_1}\frac{1}{s_1^{1+\mu}s_2^{1+\mu}}\int\mathrm{d}a_1\mathrm{d}a_2H(a_1^{1/\mu})H(a_2^{1/\mu})A_2a_1^{2/\mu}\ .
\end{aligned}\tag{93}$$

We note that only the second term of (92) contains the correlations between the τ_k. Thus, the large time scaling of the covariance of the number of distinct sites visited is not affected by the correlations between the τ_k if the first term dominates the second one,

$$s_1s_2\ \mathrm{Cov}\left[\sum_{k=1}^{n_1-1}\tau_k,\sum_{k=n_1}^{n_2-1}\tau_k\right]=O\left(s_1s_2n_1^{2/\mu}\right)\tag{94}$$

or in other words, for any value of n_2

$$\mathrm{Cov}\left[\sum_{k=0}^{n_1-1}\tau_k,\sum_{k=n_1}^{n_2-1}\tau_k\right]=O\left(n_1^{2/\mu}\right)\ .\tag{95}$$

Thus, using the Tauberian theorem, under the condition (95) , we obtain (85). We verify this relation in Supplementary Figure 14 as well as the hypothesis in Supplementary Figure 15. Even though there is evidence that τ_k have algebraically decreasing correlations, since the condition (95) is respected, the time behavior of the covariance still holds. We emphasize that in the case in which this criterion is not respected, the covariance between distinct sites visited has still a lower bound given by (81), as we show for the T-tree in Supplementary Figure 16. Despite the noisiness of the data for $\frac{1}{n_1^{2\mu}}\mathrm{Cov}\left[\sum_{k=0}^{n_1-1}\tau_k,\sum_{k=n_1}^{n_2-1}\tau_k\right]$, we observe that the covariance does not respect (95). This is why the two-time covariance of the number of distinct sites visited is not the same as that for Lévy flights.

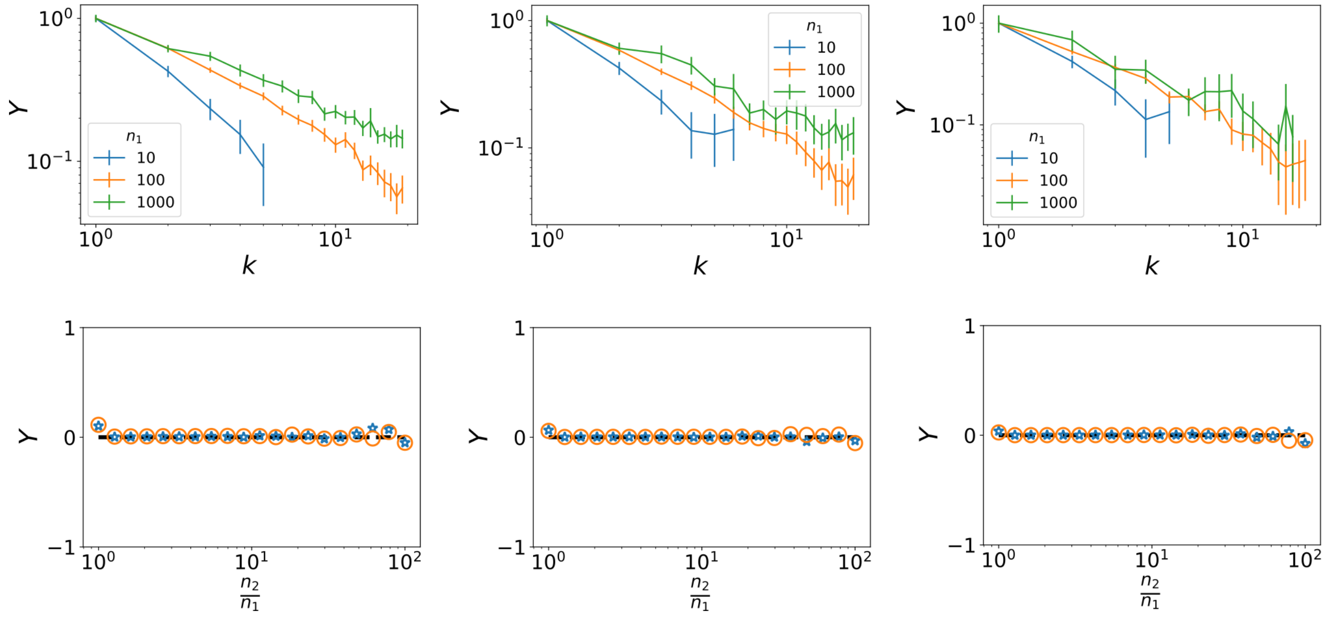

Supplementary Figure 15. **Correlation functions of $1d$ Lévy flights' τ_k:**
Upper: Computation of the covariance $Y = \mathrm{Cov}[\tau_{n_1}, \tau_{n_1+k}]/\mathrm{Var}(\tau_{n_1})$ as a function of k. Errors bars are the 95% confidence intervals.
Lower: Summed covariance divided by $n_1^{2\alpha}$, $Y \equiv \frac{1}{n_1^{2\alpha}}\mathrm{Cov}\left[\sum_{k=0}^{n_1-1}\tau_k, \sum_{k=n_1}^{n_2-1}\tau_k\right]$ as a function of n_2 for different values of n_1 (in blue $n_1 = 10$, in orange $n_1 = 100$ and in green $n_1 = 1000$).
The α values are $\alpha = 1.3$ (left), $\alpha = 1.5$ (middle) and $\alpha = 1.7$ (right).

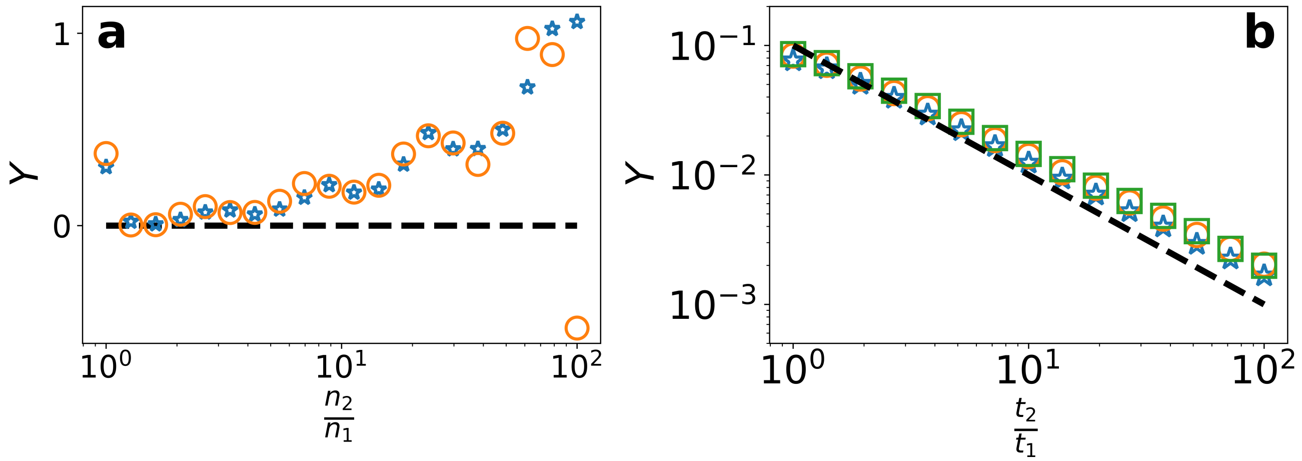

Supplementary Figure 16. **Correlation functions of the T-tree: a** $Y = \frac{1}{n_1^{2/\mu}}\mathrm{Cov}\left[\sum_{k=1}^{n_1}\tau_k, \sum_{k=n_1+1}^{n_2}\tau_k\right]$ as a function of n_2 ($n_1 = 10$ in blue stars, $n_1 = 100$ in orange circles). **b** $Y = \frac{\mathrm{Cov}[N(t_1),N(t_2)]}{\mathbb{E}(N(t_1))\mathbb{E}(N(t_2))}$ compared to a function proportional to $\frac{t_1}{t_2}$ ($t_1 = 10$, 100, and 1000 represented by blue stars, orange circles and green squares, respectively).

However, it still gives a lower bound on the scaling of the covariance. Thus for any recurrent random walk, the covariance is long range. For lattice random walks [S26] and recently for α-stable random walks [S27] that satisfy $\mu > 3/2$, it has been shown that the process $N(t) - \mathbb{E}(N(t))$ converges to a Brownian motion of variance $\mathrm{Var}(N(t))$ from which we deduce the covariance. Indeed, noting $\sigma = \lim_{t\to\infty}\frac{\sqrt{\mathrm{Var}(N(t))}}{\sqrt{t}}$ and $(B_t)_{t\geq 0}$ the brownian motion such that

$\text{Cov}\left[B_{t_1}, B_{t_2}\right] = \min\left(t_1, t_2\right)$, we have that

$$\begin{aligned}\text{Cov}\left[N(t_1), N(t_2)\right] &\approx \sigma^2 \text{Cov}\left[B_{t_1}, B_{t_2}\right] \\ &= \sigma^2 \min\left(t_1, t_2\right)\end{aligned} \tag{96}$$

The case $1 \leq \mu \leq 3/2$ remains an open question.

C. Non-Markovian RWs

1. CTRW

In this section, we show how the formalism of the main text, presented in the case of RWs in discrete time, can be extended to cover the important case of Continuous Time Random Walks (CTRWs).
For such walks, we notice that having the statistics of τ_n in the discrete setting (noted F_n) is enough to get the statistics $\tilde{\tau}_n$ in the continuous setting (noted F_n^{CTRW}), as adding the waiting times between jumps do not modify the statistics of the set of n visited sites. By defining λ_k as the time associated to the kth displacement of the random walker:

$$F_n^{\text{CTRW}}(t) = \sum_{k=1}^{\infty} F_n(k) \mathbb{P}(\lambda_1 + \ldots + \lambda_k = t) \; . \tag{97}$$

We will be mainly interested in the case where $\lambda_k' s$ are i.i.d. with power law tails, $p(\lambda_k \geq t) \propto t^{-\beta}$ at large time, with $\beta < 1$. They represent waiting times with infinite average. To simplify the calculations, we will assume that λ_k are drawn from a Lévy stable law of parameter β such that $\lambda_1 + \ldots + \lambda_k$ and $k^{1/\beta}\lambda_0$ have the same distribution. We compute the scaling behaviours of the distribution of $\tilde{\tau}_n$ in the following manner for the recurrent and marginally recurrent random walks:

$$\begin{aligned}\mathbb{P}(\tilde{\tau}_n \geq t) &\approx \sum_{k=1}^{t_n} \frac{A}{k^{1+\mu}} \mathbb{P}(k^{1/\beta}\lambda_0 \geq t) \\ &\approx \sum_{k=1}^{\min(t_n, t^\beta)} \frac{A}{k^{1+\mu}} \frac{Bk}{t^\beta} + \sum_{k=\min(t_n, t^\beta)}^{t_n} \frac{A}{k^{1+\mu}}\end{aligned} \tag{98}$$

where we have been using that for Lévy stable laws, the tail distribution is $\sim \frac{B}{t^\beta}$ for $t \gg 1$. We neglect the exponential and stretched exponential regimes occurring at $k > t_n$. We consider first the case, $t^\beta > t_n$:

$$F_n^{\text{CTRW}}(t) \propto -\frac{\mathrm{d}}{\mathrm{d}t}\left(\sum_{k=1}^{t_n} \frac{A}{k^{1+\mu}} \frac{Bk}{t^\beta}\right) \propto \begin{cases} \frac{t_n^{1-\mu}}{t^{\beta+1}} \propto \frac{n^{1/\mu - 1}}{t^{\beta+1}} & \text{if } \mu < 1 \\ \frac{\ln t_n}{t^{\beta+1}} \propto \frac{\ln n}{t^{\beta+1}} & \text{if } \mu = 1 \; . \end{cases} \tag{99}$$

while for $t^\beta < t_n$,

$$F_n^{\text{CTRW}}(t) \propto -\frac{\mathrm{d}}{\mathrm{d}t}\left(\sum_{k=1}^{t^\beta} \frac{A}{k^{1+\mu}} \frac{Bk}{t^\beta} + \sum_{k=t^\beta}^{t_n} \frac{A}{k^{1+\mu}}\right) \propto \begin{cases} \frac{1}{t^{\beta\mu+1}} & \text{if } \mu < 1 \\ \frac{\ln t}{t^{\beta+1}} & \text{if } \mu = 1 \; . \end{cases} \tag{100}$$

We check this regime in Supplementary Figure 17 for different values of μ and β. The distribution F_n^{CTRW} of $\tilde{\tau}_n$ is always algebraic, but the exponent changes at $t = t_n^{1/\beta}$: it is first algebraic of power $-1 - \beta\mu$, and then of power $-\beta - 1$ as the distribution of the waiting times. It results from a competition between the algebraic waiting times and the algebraic distribution of τ_n. Adding one additional waiting time has an algebraic cost. However, when t^β is large compared to the cut-off time t_n, adding one waiting time has an exponential cost: thus, it is more likely to obtain large exit time because one waiting time is large than because τ_n is large. Thus, the exponential and stretched

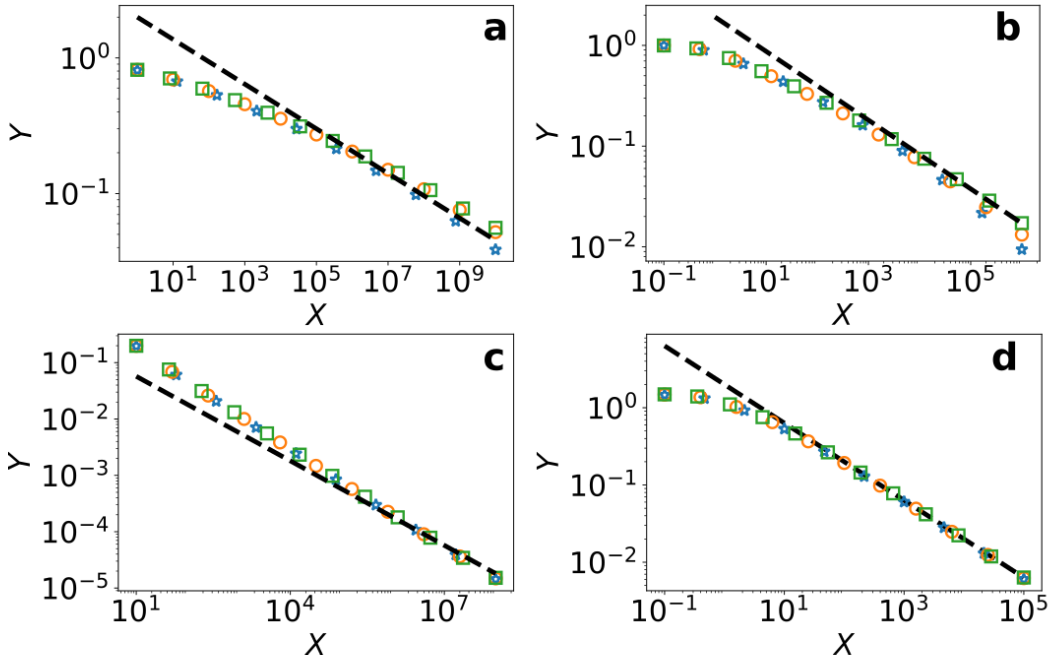

Supplementary Figure 17. **CTRW of recurrent nearest neighbour RWs: a** on percolation clusters, $\beta = 0.25$, **b** on a Sierpinski gasket, $\beta = 0.25$, **c** on a $2d$ square lattice, $\beta = 0.5$, **d** on a a $3d$ hypercubic lattice, $\beta = 0.5$. Each tail distribution $Y = \mathbb{P}(\tilde{\tau}_n > t)$ is compared to the predicted exponent of (99) against $X = t$. For the marginal case **c**, $Y = \mathbb{P}(\tilde{\tau}_n > t)/\ln t$.

exponential regimes play no role in the algebraic law. This is why for transient walks, $\mu > 1$, the law of $\tilde{\tau}_n$ is simply algebraic of parameter $-1-\beta$ as can be seen in Supplementary Figure 17: only a few values of τ_n matter. It is much more likely to obtain a large value of $\tilde{\tau}_n$ because one waiting time dominates than because τ_n is large. We can thus generalize our result to CTRWs (in particular for $\beta < 1$) by switching the exponent $1+\mu$ of Markovian RWs to $1+\beta\mu$, which translates into $1+\beta(1-\theta/\beta)$ when the algebraic decay is expressed in terms of the persistence exponent θ.

2. 1*d* *Lévy flights with crossing*

In this section, we show how the formalism of the main text, presented in the case of Markovian RWs for which a renewal type equation between the propagator and the first-passage time density holds, can be extended to cover the important case of $1d$ Lévy flights that find sites with the so-called "crossing" convention (to be opposed with the "arrival" condition, used implicitly in the main text when referring to Lévy flights). While the renewal equation holds for Lévy flights with the arrival condition, it does not for Lévy flights with the crossing condition. Here we propose a direct analysis of the $1d$ Lévy flights with the crossing condition. Importantly, we show that our results (and in particular the exponent of the algebraic decay) still apply in this case.

We consider a Lévy flight, for which every step is drawn from a Zif's law $p(l) = \frac{1}{2\zeta(1+\alpha)|l|^{1+\alpha}}$, and contrary to what we presented in the main text Fig. 3**a** of the main text, all sites the walker flies above are visited (the visited region contains no holes). Thus, the analysis on τ_n can be separated in two parts:

- The walker is 'flying' when it visits the nth site: in this case, $\tau_n = 0$.
- The walker stopped exactly at the last visited site n. Thus, the time distribution of exit times is given by the exit time distribution of a Lévy flight from an interval of size n, starting at distance 1 from the border. We write this distribution as $\tilde{F}_n(t)$.

We illustrate this definition in Supplementary Figure 18. We define p_n as the probability for the walk to stop exactly at the nth visited site. Thus the distribution F_n of τ_n can be described as

$$F_n(t) = \delta(t)(1-p_n) + p_n\tilde{F}_n(t)\ . \tag{101}$$

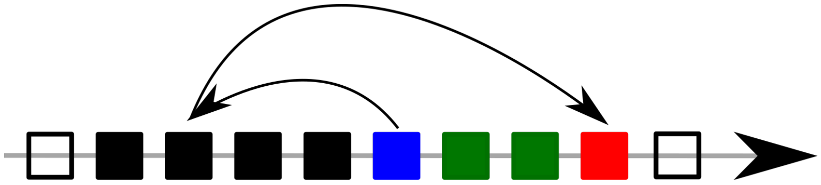

Supplementary Figure 18. We consider the example where 5 sites have been visited (4 black squares and 1 blue where the RW starts). The Lévy flight performs two jumps before arriving on the red site it never visited before. During the second flight, the walker flew above two other previously unvisited sites (in green). Thus, $\tau_5 = 2$, $\tau_6 = 0$, and $\tau_7 = 0$. τ_8 is given by the first exit time from the interval of 8 squares starting from the red one.

This results in

$$\langle \tau_n^k \rangle \sim p_n \int_0^\infty t^k \tilde{F}_n(t) \mathrm{d}t \, . \tag{102}$$

We use the result from [S7] for the distribution of the first hitting time of a target at distance $r = 1$ in a line of length $R = n/2$ with a reflecting boundary condition. It is indeed equivalent to the first hitting time of one boundary of a finite domain of length n starting at distance one from one border. From this, we get that

$$\int_0^\infty t^k \tilde{F}_n(t) \mathrm{d}t \sim n^{k\alpha - \alpha/2} \, . \tag{103}$$

Next, we derive the scaling with n of the first moment of τ_n from the scaling with t of $N(t)$. $N(t)$ is given by the difference between the maximum and the minimum of the position of the Lévy flights after t steps, which are know to scale as $t^{1/\alpha}$ [S28]. We note that for $\alpha < 1$, the average of $N(t)$ is infinite, but the average of $N(t)^q$ for q small enough is finite and given by $t^{q/\alpha}$. This is why in the following we replace $N(t)$ by $t^{1/\alpha}$ for any value of α. By using the approximate relation

$$\frac{\mathrm{d}N(t)}{\mathrm{d}t} \propto \frac{1}{\langle \tau_{N(t)} \rangle} \tag{104}$$

we obtain that

$$\langle \tau_n \rangle \sim n^{\alpha - 1} \sim p_n n^{\alpha/2} \, . \tag{105}$$

It implies that for any value of α,

$$p_n \sim n^{\alpha/2 - 1} \, , \tag{106}$$

which we check on the figure Supplementary Figure 19. Combining (102), (103) and (106), we get

$$\langle \tau_n^k \rangle \sim n^{k\alpha - 1} \, . \tag{107}$$

Besides, using (101) and (106), we have that τ_n follows a scaling behaviour in the limit $t, n \to \infty$, t/n^α fixed:

$$F_n(t) \sim \frac{1}{n^{1+\alpha}} \psi\left(t/n^\alpha\right) \, , \tag{108}$$

where ψ is algebraic at small $x = t/n^\alpha$, of exponent $-1 - \theta = -1 - 1/2$ where θ is the persistence exponent [S7],

$$\psi(x) \sim x^{-1-1/2} \, . \tag{109}$$

In particular, the formula given in Table 1 of the main text for the exponent of the algebraic decay at early times is valid in the case of this non-Markovian random walk.

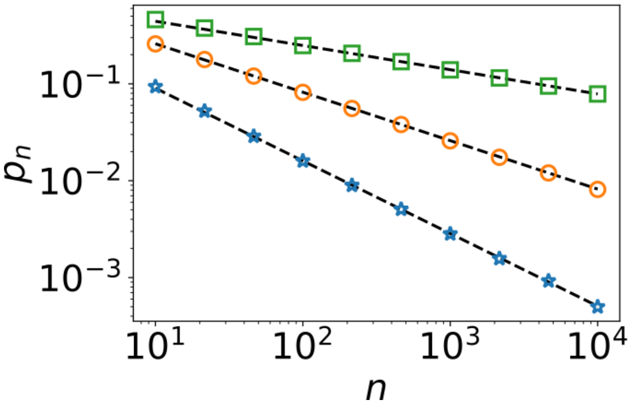

Supplementary Figure 19. p_n for $\alpha = 0.5$, $\alpha = 1$ and $\alpha = 1.5$ (blue stars, orange circles, and green squares, respectively) compared to their predicted dependence on n, $n^{\alpha/2-1}$.

3. Lévy walks

In this section, we extend the result of Lévy flights with crossings to describe the Lévy walk. From the result on Lévy flights with crossings, we deduce the behaviour of τ_k for Lévy walks, where the jumps are not instantaneous but have a duration equal to the length of the jump. For $\alpha > 1$, the result is unchanged because the waiting times have finite mean, but for $\alpha < 1$, by applying what we did in S5.C.*1* for CTRW to our situation, we obtain that the early-time regime is still algebraic but with a modified exponent (here, $\beta \to \alpha$ and $\mu \to 1/2$),

$$F_n(t) \sim \frac{1}{n^2}\phi\left(t/n\right) \ , \tag{110}$$

where ϕ is algebraic at small $x = t/n$, of exponent $-1-\alpha/2$

$$\phi(x) \sim x^{-1-\alpha/2} \ . \tag{111}$$

Once again, we find that the exponent of Table 1 is still valid for this other type of non-Markovian random walk, which strongly confirms the exponent $1+\beta(1-\theta/\beta)$ (for CTRW with infinite waiting times, $\beta < 1$, and otherwise $\beta = 1$).

4. Algebraic decay for non-Markovian recurrent random walks

Because of the applicability of our result to the non-Markovian cases of Lévy flights with crossing and Lévy walks for any value of α in $1d$, we test the exponent $2-\theta$ (for walks with waiting times of finite mean) of the algebraic decay of the distribution $F_n(\tau)$ in three other cases: the sub-diffusive Fractional Brownian Motion (characterized by the Hurst exponent H such that $d_\mathrm{w} = 1/H > 2$), the super-diffusive Fractional Brownian Motion ($d_\mathrm{w} = 1/H < 2$) and the True Self Avoiding Random Walk [S29] ($d_\mathrm{w} = 3/2$). We simulate the Fractional Brownian motion by using the module fbm [S30] of python based on Hosking's method [S31]. We discretize the line in intervals of size one, and consider that an interval has been visited when the RW enters it for the first time. τ_n is the time elapsed between visit of the nth interval and the new $(n+1)^{\mathrm{st}}$ interval. For the True Self Avoiding Random Walk, we record the number of visits C_i of site i. The probability to jump to the site on the right is given by $\exp(-C_{i+1})/(\exp(-C_{i+1})+\exp(-C_{i-1}))$, otherwise the RW jumps on the left. We argue that $2-\theta$ is valid for a large range of non-Markovian RWs, despite the fact that the renewal equation we used to obtain this exponent is not valid anymore.

In Fig. 4**g**, **h** and **i** of the main text, we show the algebraic decay of the probability $F_n(\tau)$ for the True Self Avoiding Walk and the Fractional Brownian Motion. Each is successfully compared to the algebraic decay $\tau^{-1-\mu}$ where $\mu = \frac{d_\mathrm{f}}{d_\mathrm{w}}$. Based on the previous results for the Markovian and the non-Markovian RWs, we make the following prediction that for any recurrent random walks, the algebraic exponent governing the early time regime is given by

$$F_n(\tau) \propto 1/\tau^{1+\beta-\theta} \tag{112}$$

where θ is the persistent exponent, and β is either 1 or the exponent of the algebraic decay of the waiting time distribution of a jump $p(\lambda_k \geq t) \propto t^{-\beta}$ if this exponent is smaller than 1.
For discrete (in time) Markovian random walks, $\mu = \frac{d_f}{d_w} = 1 - \theta$ and $\beta = 1$: we get back to the exponent $-1-\mu$ predicted in S3.A. For CTRW on Markovian RWs, $\theta = \beta(1-\mu)$ and we get back to (100). For $1d$ Lévy flights with crossing, $\theta = 1/2$ and $\beta = 1$ while for Lévy walks $\theta = \alpha/2$ and $\beta = \alpha$. We obtain again (109) and (111). Finally, for True Self Avoiding Walks and Fractional Brownian motion $\theta = 1 - \mu$ and $\beta = 1$ which gives again the exponent we confirm numerically in Fig. 4 of the main text.

SUPPLEMENTARY REFERENCES

[S1] S. Redner, *A guide to first-passage processes* (Cambridge University Press, 2001) pp. 43–45.
[S2] B. D. Hughes, *Random Walks and Random Environments* (Clarendon Press: Oxford University Press, Oxford: New York, 1995).
[S3] J. E. Gillis and G. H. Weiss, Expected Number of Distinct Sites Visited by a Random Walk with an Infinite Variance, J. Math. Phys. **11**, 1307 (2003).
[S4] A. Dembo, Y. Peres, and J. Rosen, How large a disc is covered by a random walk in n steps?, Ann. Probab. **35**, 577 (2007).
[S5] The prefactor $1/e^2$ is here only to ease comparison with numerical simulations.
[S6] S. Condamin, V. Tejedor, R. Voituriez, O. Bénichou, and J. Klafter, Probing microscopic origins of confined subdiffusion by first-passage observables, Proc. Natl. Acad. Sci. USA **105**, 5675 (2008).
[S7] N. Levernier, O. Bénichou, T. Guérin, and R. Voituriez, Universal first-passage statistics in aging media, Phys. Rev. E **98**, 022125 (2018).
[S8] P. Erdős and P. Révész, Three problems on the random walk in $\mathbb{Z}_d$, Stud. Sci. Math. Hung. **26**, 309 (1991).
[S9] P.-G. de Gennes, *Scaling Concepts in Polymer Physics* (Cornell University Press, 1979).
[S10] A. Y. Grosberg and A. R. Khokhlov, *Statistical Physics of Macromolecules* (AIP Press, New York, 1994).
[S11] D. Plyukhin and A. V. Plyukhin, Random walks with fractally correlated traps: Stretched exponential and power-law survival kinetics, Phys. Rev. E **94**, 042132 (2016).
[S12] M. Kac, On the notion of recurrence in discrete stochastic processes, Bull. Am. Math. Soc. **53**, 1002 (1947).
[S13] F. Van Wijland and H. Hilhorst, Universal fluctuations in the support of the random walk, J. Stat. Phys. **89**, 119 (1997).
[S14] A. Mariz, F. van Wijland, H. Hilhorst, S. Gomes Júnior, and C. Tsallis, Statistics of the one-dimensional riemann walk, J. Stat. Phys. **102**, 259 (2001).
[S15] J. Klafter and I. M. Sokolov, *First Steps in Random Walks: From Tools to Applications* (Oxford University Press, Oxford, UK, 2011).
[S16] S. Wolfram, *A New Kind of Science* (Wolfram Media, Inc., 2002) Chap. 5.4.
[S17] I. Majid, D. B. Avraham, S. Havlin, and H. E. Stanley, Exact-enumeration approach to random walks on percolation clusters in two dimensions, Phys. Rev. B **30**, 1626 (1984).
[S18] F. Wang and D. P. Landau, Efficient, multiple-range random walk algorithm to calculate the density of states, Phys. Rev. Lett. **86**, 2050 (2001).
[S19] D. P. Landau, S.-H. Tsai, and M. Exler, A new approach to monte carlo simulations in statistical physics: Wang-landau sampling, Am. J. Phys. **72**, 1294 (2004).
[S20] S. T. Tokdar and R. E. Kass, Importance sampling: a review, Wiley Interdisciplinary Reviews: Computational Statistics **2**, 54 (2010).
[S21] G. Barkema, P. Biswas, and H. van Beijeren, Diffusion with random distribution of static traps, Phys. Rev. Lett. **87**, 170601 (2001).
[S22] N. Metropolis, A. W. Rosenbluth, M. N. Rosenbluth, A. H. Teller, and E. Teller, Equation of state calculations by fast computing machines, J. Chem. Phys. **21**, 1087 (1953).
[S23] F. van Wijland, S. Caser, and H. J. Hilhorst, Statistical properties of the set of sites visited by the two-dimensional random walk, J. Phys. A: Math. Gen. **30**, 507 (1997).
[S24] L. Régnier, M. Dolgushev, S. Redner, and O. Bénichou, Complete visitation statistics of one-dimensional random walks, Phys. Rev. E **105**, 064104 (2022).
[S25] J.-F. L. Gall and J. Rosen, The Range of Stable Random Walks, Ann. Probab. **19**, 650 – 705 (1991).
[S26] N. C. Jain and W. E. Pruitt, Further limit theorems for the range of random walk, J. d'Analyse Math. **27**, 94 (1974).
[S27] W. Cygan, N. Sandrić, and S. Šebek, Functional clt for the range of stable random walks, Bull. Malays. Math. Sci. Soc. **44**, 1371 (2021).
[S28] J.-P. Bouchaud and A. Georges, Anomalous diffusion in disordered media: statistical mechanisms, models and physical applications, Phys. Rep. **195**, 127 (1990).
[S29] A. Barbier-Chebbah, O. Benichou, and R. Voituriez, Anomalous persistence exponents for normal yet aging diffusion, Phys. Rev. E **102**, 062115 (2020).
[S30] C. Flynn, Exact methods for simulating fractional brownian motion and fractional gaussian noise in python, "`https://github.com/crynn/fbm`" (2007).

[S31] J. R. M. Hosking, Modeling persistence in hydrological time series using fractional differencing, Water Resour. Res. **20**, 1898–1908 (1984).
